# Marine protected areas, marine heatwaves, and the resilience of nearshore fish communities

**DOI:** 10.1038/s41598-023-28507-1

**Published:** 2023-01-25

**Authors:** Shelby L. Ziegler, Jasmin M. Johnson, Rachel O. Brooks, Erin M. Johnston, Jacklyn L. Mohay, Benjamin I. Ruttenberg, Richard M. Starr, Grant T. Waltz, Dean E. Wendt, Scott L. Hamilton

**Affiliations:** 1 Moss Landing Marine Laboratories, San Jose State University, Moss Landing, CA 95039 USA; 2 Odum School of Ecology, University of Georgia, Athens, GA 30602 USA; 3 Department of Marine Science, California State University Monterey Bay, Seaside, CA 93955 USA; 4 Center for Coastal Marine Sciences, Biological Sciences Department, California Polytechnic State University, San Luis Obispo, CA 93407 USA

**Keywords:** Biodiversity, Climate-change ecology, Community ecology, Conservation biology

## Abstract

Anthropogenic stressors from climate change can affect individual species, community structure, and ecosystem function. Marine heatwaves (MHWs) are intense thermal anomalies where water temperature is significantly elevated for five or more days. Climate projections suggest an increase in the frequency and severity of MHWs in the coming decades. While there is evidence that marine protected areas (MPAs) may be able to buffer individual species from climate impacts, there is not sufficient evidence to support the idea that MPAs can mitigate large-scale changes in marine communities in response to MHWs. California experienced an intense MHW and subsequent El Niño Southern Oscillation event from 2014 to 2016. We sought to examine changes in rocky reef fish communities at four MPAs and associated reference sites in relation to the MHW. We observed a decline in taxonomic diversity and a profound shift in trophic diversity inside and outside MPAs following the MHW. However, MPAs seemed to dampen the loss of trophic diversity and in the four years following the MHW, taxonomic diversity recovered 75% faster in the MPAs compared to reference sites. Our results suggest that MPAs may contribute to long-term resilience of nearshore fish communities through both resistance to change and recovery from warming events.

## Introduction

Fluctuating ocean conditions due to anthropogenic climate change can affect individual species, community structure, and ecosystem function. Extreme climatic events have altered a range of ecosystem processes, including increasing direct mortality by exceeding species’ physiological thresholds^1^, altering biogeochemical cycles^2^, creating phenological mismatches with food resources^3^, and shifts in species ranges^4,5^. Changes in these processes have led to rapid reorganization of marine communities^6,7^, loss of ecosystem function, and increasing exploitation of certain fish species from human harvest^8^. Systems that are already degraded or stressed from other anthropogenic impacts may not be able to withstand or resist the effects of thermal disturbances or other climatic events. For instance, overfishing can reduce the resilience of kelp forest ecosystems to respond to climate induced phase shifts by altering trophic structure and key species interactions^9,10^. Marine protected areas (MPAs) can possibly mitigate the compounding effects of over exploitation and climate change, however their performance in the face of ocean warming events remains uncertain.

Marine heatwaves (MHWs) are intense thermal anomalies where water temperatures are elevated above the 90th percentile of the long-term average for more than 5 days^11^. Climate projections predict that the frequency, severity, and duration of MHWs will increase in the coming decades^12^, with synthetic analyses indicating generally deleterious effects for foundation species and ecosystem function in all ocean basins^13^. Modeling efforts suggest that MHWs can cause decreases in fish biomass and range shifts in target fish stocks at least four times faster than the effects of decadal-scale temperature change alone^14^. Empirical work has documented mass mortality events across a wide range of taxa around the globe^15^ and major changes in fish populations and communities following MHWs, confirming many theoretical predictions. For example, Wernberg et al.^16^ reported that anomalous warming events led to a reduction in habitat-forming seaweed abundance with cascading effects to the fish and invertebrate community, whereby the community became dominated by fishes with warm-water affinities. Additionally, MHWs appear to cause increased juvenile fish mortality at lower latitudes resulting in poleward shifts of certain populations^17^ and potentially altering adult community composition for years following anomalous temperature events.

Between October 2014 and June 2016, California experienced an intense MHW coined “the Blob”, followed by a large El Niño Southern Oscillation (ENSO) event, where ocean temperature anomalies were persistently elevated along the entire U.S. West Coast by up to 6 °C^18^ and down to depths of 140 m^19^. The MHW caused drastic changes in marine ecosystems from the Gulf of Alaska, USA to Baja California, Mexico^20,21^, triggering cascading effects on oceanographic processes and conditions such as net primary production (NPP; Ref.^22^. Warming events are relatively common in the California Current Ecosystem (CCE), a wind-driven coastal upwelling system^23^; Thompson et al.^24^ reported that over 225 heatwaves occurred in the region between 1982 and 2019. However, the unusually long duration and large spatial extent of the 2014–2016 MHW may have had greater effects on marine communities than previous and subsequent warm water events along the CCE. A recent study using long-term data from the Gulf of Alaska found that the negative effects (e.g. decreased abundance, biomass, reproductive success) of the 2014–2016 MHW on a variety of species (e.g. birds, fishes, marine mammals) persisted for over 2 years following a return to normal water temperatures^25^, indicating that the impacts of MHWs may persist for many years.

A great deal of theoretical and empirical work suggests that MPAs and fully no-take marine reserves may buffer marine communities from a range of perturbations^26–28^. Furthermore, networks of marine reserves, in contrast to single or stand-alone reserves, are predicted to provide increased resistance and resilience to various anthropogenic and climatic stressors^29–31^. However, while there is evidence that MPAs may be able to buffer some individual species from climate impacts^30^, we still lack information about whether and/or how MPAs can mitigate large-scale changes in marine communities in response to MHWs, if they are in fact able to prevent community changes^32^. Additionally, MPAs may facilitate the ‘protection paradox’ by which species that are most sensitive to disturbance events benefit the most from MPA implementation. However, these species may be more vulnerable to climatic events, resulting in larger declines or community changes in MPAs following disturbances like MHWs^33^.

Along the California coast, a network of marine reserves was established between 2007 and 2012 to reduce the effects of overfishing and protect overall ecosystem health^34,35^, providing an opportunity to explore the impacts and recovery of a MHW on nearshore marine communities. Past studies have shown that fish biomass increases rapidly inside MPAs in southern California^36,37^ and that MPAs can help resist biological invasion^29^. While those same MPAs are not immune to community change in response to MHWs^32^, it is unknown how upwelling dominated ecosystems (e.g. the California Current Ecosystem) will respond or recover following warm water events.

Here we evaluate the influence of the 2014–2016 MHW on recreationally and commercially targeted fish communities inside and outside of four no-take MPAs sampled along the central coast of California from 2007 to 2020. We asked 4 main questions: (1) Did fish diversity and community composition respond to the MHW similarly in MPAs and in areas open to fishing? (2) Did the proportional representation of individual species and/or functional groupings in the community change in relation to the MHW? (3) Did fish assemblages in MPAs and areas open to fishing exhibit similar responses to the MHW? and (4) How did MPAs influence how individual species and the broader assemblage recover from the MHW? Using long-term monitoring data collected by the community-based California Collaborative Fisheries Research program (CCFRP)^38^, we examined biodiversity, community structure, and species-specific trends in MPAs and areas open to fishing before (2007–2013), during (2014–2016) and after (2017–2020) the MHW. We hypothesized that community structure would shift greatly both inside and outside MPAs, resulting in novel communities following the MHW^16,32^. In addition, we expected communities to differ inside and outside of MPAs during the MHW, and that communities inside marine reserves would be more resistant to changes and would recover faster from the MHW than in areas open to fishing.

## Results

### Environmental conditions

**Figure 1:**
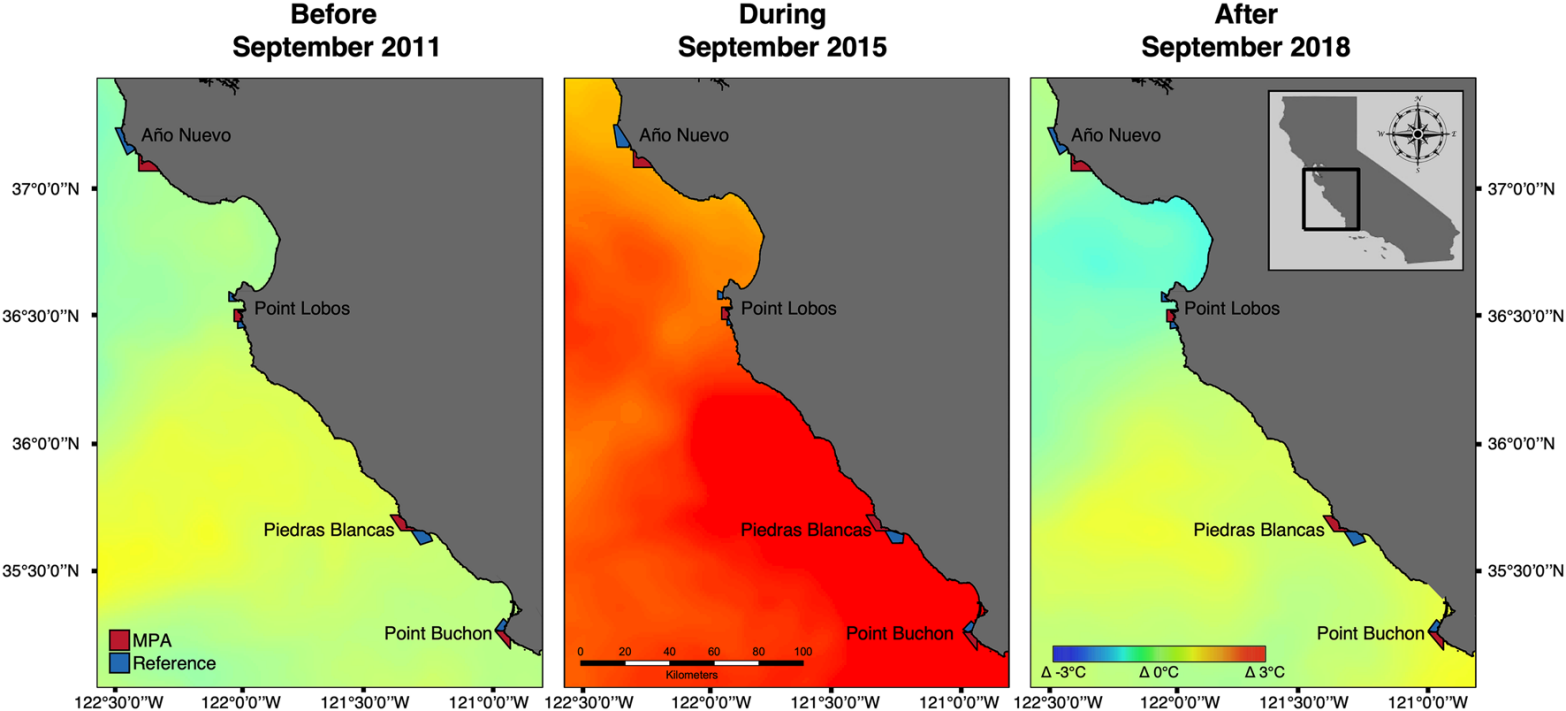
Maps of thermal anomalies in Central California in response to the marine heatwave (MHW). CCFRP MPA and reference sites overlaid with sea surface temperature anomalies for time periods before during and after the 2014–2016 MHW. All temperature anomalies were extracted as monthly means for the month of September three years prior to the heatwave (2011), during the heatwave (2015), and two years post-heatwave (2018). The years selected to represent the pre- and post-MHW were chosen due to presence of non-anomalous conditions during the summer months (MOCI = − 0.67 and 0.01, respectively). Data were extracted from the NOAA ERDDAP Multi-scale Ultra-high Resolution (MUR) SST Analysis Anomaly dataset (https://coastwatch.pfeg.noaa.gov/erddap/index.html) and maps were constructed in ArcMap (Version 10.8.1; https://desktop.arcgis.com/en/arcmap/). An extended version of this figure with all sampling years can be found in Fig. S1.

During the peak of the MHW in September 2015, water temperatures were approximately 3 °C warmer than historical conditions across all sites (Fig. 1; Supplementary Fig. S1). Overall, there were clear differences in temperature (SST) and primary production (NPP) before, during, and after the marine heatwave and across the four broad geographic locations, but no interaction between the two. Both mean SST (ANOVA: MHW: F_2,100_ = 67.6, p < 0.001, location: F_3,100_ = 16.76, p < 0.001) and maximum SST (ANOVA: MHW: F_2,100_ = 32.43, p < 0.001, location: F_3,100_ = 4.21, p = 0.008) were highest at the most southern location (Point Buchon). During non-heatwave years, mean monthly water temperatures ranged from 9.8 °C to 15.8 °C and during the heatwave years (2014–2016) mean monthly temperatures ranged from a low of 11.3 °C to peak temperatures of 17.5 °C (Supplementary Fig. S2). NPP was variable across all locations and years, as is expected in this dynamic upwelling ecosystem. NPP was on average lower prior to the heatwave and higher following the heatwave (ANOVA: MHW: F_2,92_ = 7.33, p = 0.001). NPP was also the highest on average at Año Nuevo compared to the other locations (ANOVA location: F_3,92_ = 9.90, p < 0.001). Both wind speed and significant wave height varied across locations (ANOVA wind speed: F_3,52_ = 27.96, p < 0.001, wave height: F_3,100_ = 12.50, p < 0.001) but did not vary in relation to the MHW (ANOVA wind speed: F_2,52_ = 1.68, p = 0.19, wave height: F_2,100_ = 0.78, p = 0.46). Interestingly, both the highest wind speeds and lowest wave heights over our sampling period occurred at the Piedras Blancas locations (Supplementary Table S1).

### Taxonomic diversity

**Figure 2:**
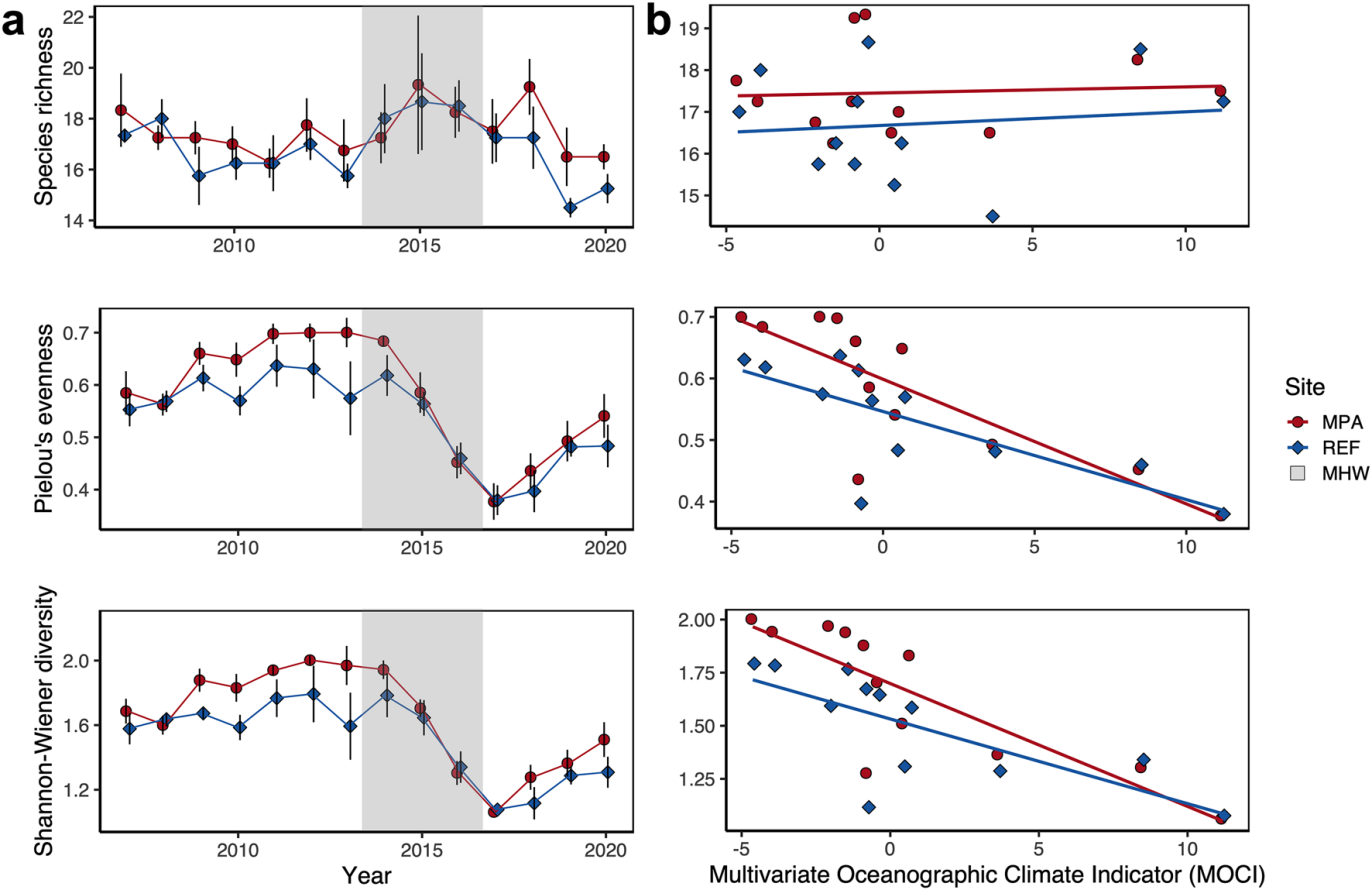
Changes in diversity metrics (species richness, Pielou’s evenness and Shannon–Wiener diversity) from 2007–2020 in relation to (**a**) the 2014–2016 MHW and (**b**) a 2-year lag in the multivariate oceanographic climate indicator (MOCI).

We found a marginally significant difference in species richness between MPAs and associated reference sites (ANOVA: F_1,102_ = 12.00, p = 0.06), with higher richness inside MPAs across all years (Fig. 2a). We also detected a difference in richness with higher richness during the MHW compared to after the MHW (ANOVA: F_2,102_ = 18.24, p = 0.05). Both Pielou’s evenness and Shannon-Weiner diversity varied by MPA status (ANOVA: F_1,102_ = 5.17, p = 0.03 and F_1,102_ = 7.00, p = 0.009, respectively) and in relation to the MHW (ANOVA: F_2,102_ = 31.94, p < 0.001 and F_2,102_ = 35.96, p < 0.001, respectively) but there was no interaction among the two. Overall, Shannon-Weiner diversity decreased dramatically after the MHW and MPAs on average had greater evenness and Shannon-Weiner diversity than reference sites (Fig. 2a).

Shannon-Weiner diversity and evenness were both higher inside MPAs than reference sites along the Central coast and were slowly increasing through time prior to the MHW (ANCOVA for Shannon-Weiner diversity: Year: F_1,50_ = 5.05, p = 0.03, MPA status: F_1,50_ = 7.69, p = 0.008; ANCOVA for evenness: year: F_1,50_ = 6.58, p = 0.01, MPA status: F_1,50_ = 6.41, p = 0.02). During the MHW, Shannon-Weiner diversity and evenness collapsed to low levels, becoming similar inside and outside the MPAs (ANCOVA for Shannon-Weiner diversity: year: F_1,18_ = 23.06, p < 0.001, MPA status: F_1,18_ = 0.39, p = 0.54; ANCOVA for evenness: year: F_1,18_ = 29.76, p < 0.001, MPA status: F_1,18_ = 0.79, p = 0.39, respectively; Fig. 2a). Following the MHW, Shannon-Weiner diversity and evenness began to increase and recover rapidly in both the MPAs and their associated reference sites (ANCOVA for Shannon-Weiner diversity: year: F_1,28_ = 12.06, p < 0.001, MPA status: F_1,36_ = 2.07, p = 0.16; ANCOVA for evenness: year: F_1,28_ = 11.89, p = 0.002, MPA status F_1,28_ = 0.744, p = 0.39, respectively; Fig. 2a). While not statistically distinct due to only 4 years of data following the MHW, the slope for change in Shannon-Weiner diversity overtime inside the MPAs was 0.14 compared to 0.08 in the associated reference sites. If these slopes hold consistent, the rate of recovery for taxonomic diversity in MPAs will be 1.75 times faster than in the associated reference sites.

There was a clear negative relationship between regional oceanographic indices (Multivariate Ocean Climate Indicator; MOCI) and both Shannon-Weiner diversity and evenness with a 2-year temporal lag (ANCOVA: F_1,20_ = 33.12, p < 0.001 and F_1,20_ = 32.43, p < 0.001, respectively; Fig. 2b), indicating that fish diversity was lowest during positive MOCI anomalies two years in the past. There were differences in Shannon-Weiner diversity and evenness among MPAs and reference sites but no clear statistical interaction among MOCI and MPA status (ANCOVA: F_1,20_ = 1.12, p = 0.30 and F_1,20_ = 0.96 p = 0.34, respectively). We also detected a positive, but weaker, relationship between species richness and MOCI with a 1-year temporal lag (ANCOVA: F_1,22_ = 12.65, p = 0.002), but no interaction among MOCI or MPA status (ANCOVA: F_1,22_ = 0.005, p = 0.90).

### Community structure

**Figure 3:**
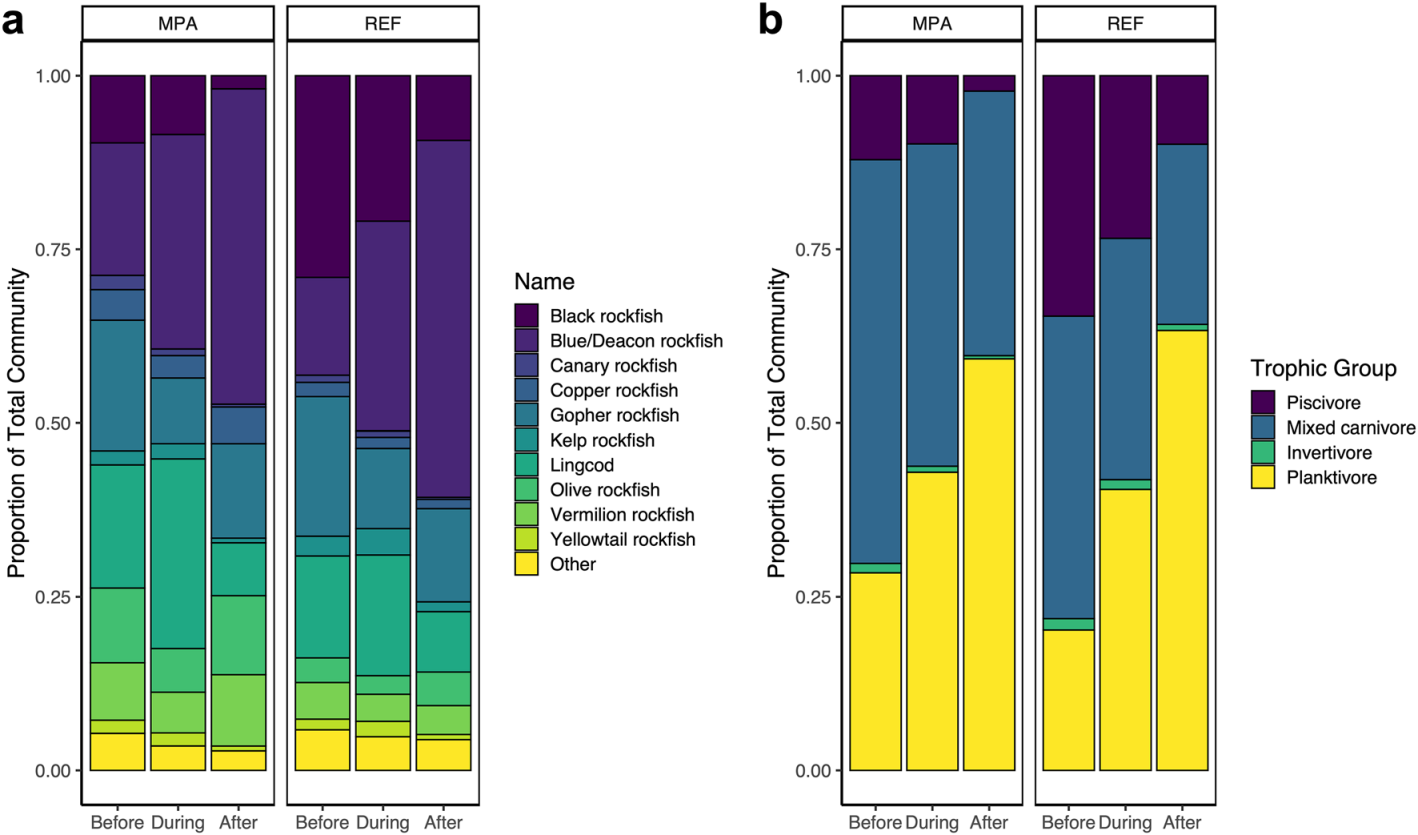
Changes in the (**a**) species community composition and (**b**) trophic structure at MPA and reference (REF) sites before (2007–2013), during (2014–2016) and after (2017–2020) the marine heatwave. In panel a, the top ten most abundant species are listed and all other species are grouped into the “Other” category.

We detected shifts in overall community composition among time periods relative to the MHW (PERMANOVA: F_2,107_ = 21.29, R^2^ = 0.26, p < 0.001) and between MPAs and associated reference sites (PERMANOVA: F_1,1107_ = 17.03, R^2^ = 0.10, p < 0.001), including a small interactive effect of the heatwave and MPA protection (PERMANOVA: F_2,107_ = 2.21, R^2^ = 0.03, p = 0.032), as communities in MPAs changed to a greater extent than communities in reference sites during and after the heatwave. Certain species changed in relative biomass after the MHW in both MPAs and reference sites. The Blue/Deacon rockfish complex and Olive rockfish comprised a much larger fraction of the community biomass following the heatwave, while Black rockfish and Lingcod comprised a smaller proportion of the community (Fig. 3a).

**Figure 4:**
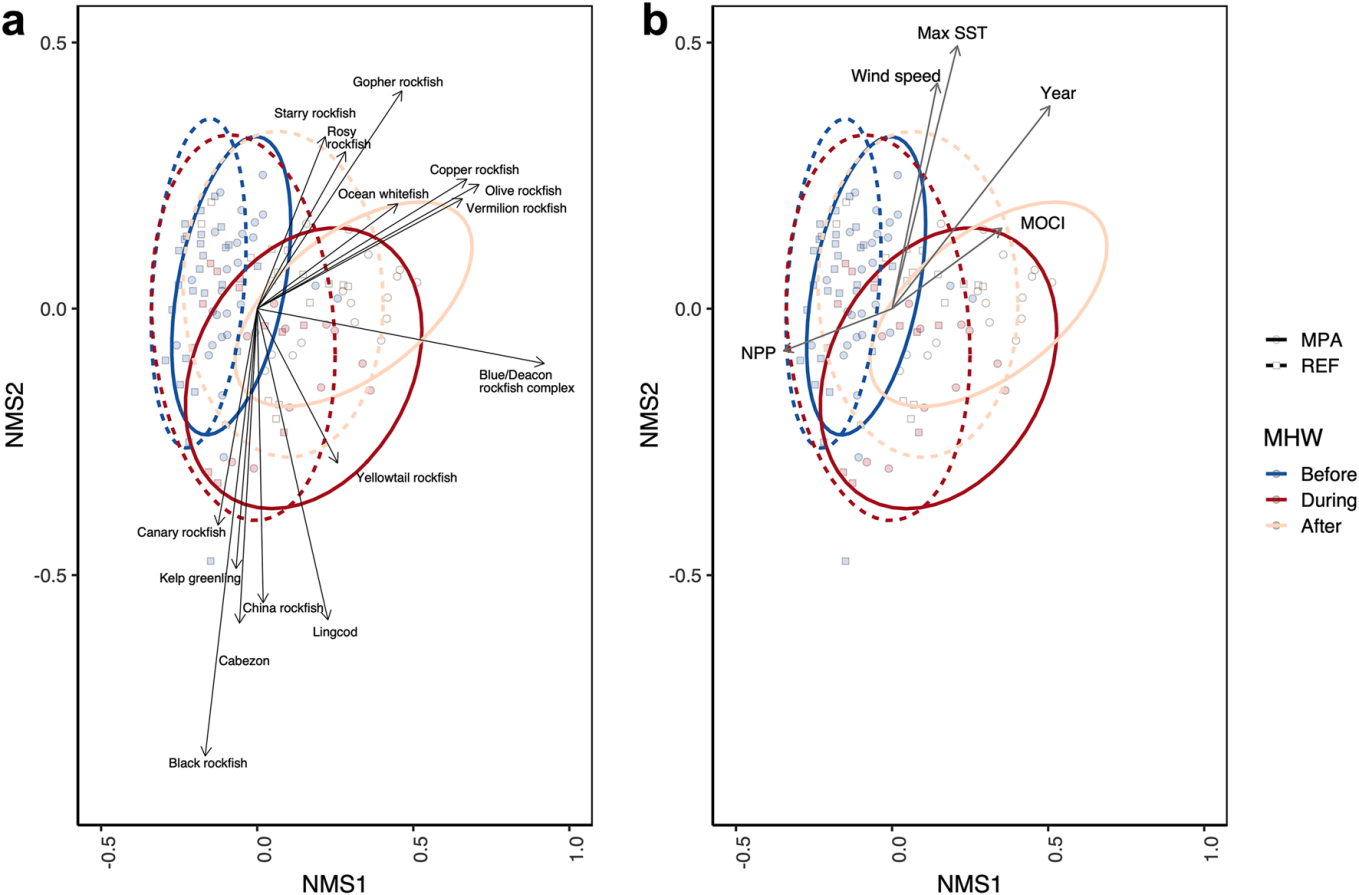
Non-metric multidimensional ordination examining the shifts in community structure with (**a**) significant species vectors and (**b**) significant environmental vectors in MPAs (solid lines) and reference (REF, dashed lines) sites before (2007–2013), during (2014–2016) and after (2017–2020) the marine heatwave (MHW). Ellipses are 95% CI.

The nMDS analysis explained at least 93% of the variation in the observed community composition (Linear R^2^ = 0.93; non-metric R^2^ = 0.98; Fig. 4). Two dimensional axes were utilized with a stress of 0.13. Both MPAs and reference sites appeared to have more similar and consistent communities before the MHW occurred. During and following the MHW, communities shifted to the right, associated with increases in maximum SST and wind speed vectors, including more planktivorous and warm water affinity species such as Ocean whitefish (*Caulolatilus princeps*; Fig. 4a and Fig. 4b). The fish community after the MHW continued to remain different from the starting conditions 4 years post-heatwave. Similar to the species composition analysis, we found the relative abundance of the Blue/Deacon rockfish complex, Olive rockfish (*Sebastes serranoides*), Black rockfish (*Sebastes melanops*), and Gopher rockfish (*Sebastes carnatus*) following the MHW were the most influential in driving the changes in community structure (SIMPERcum.contrib. = 73.2%; Table 1; Supplementary Table S2).

**Table 1:** Output of SIMPER analysis indicating the top 20 species contributing to the differences in community composition before and following the 2014–2016 MHW.

	Average contribution to dissimilarity	Standard deviation	Average abundance before MHW	Average abundance during MHW	Average abundance following MHW	Cumulative contribution
**Blue/Deacon rockfish complex**	**0.24**	**0.13**	**0.56**	**2.17**	**4.04**	**0.449**
Black rockfish	0.05	0.07	0.59	0.93	0.35	0.550
**Gopher rockfish**	**0.05**	**0.05**	**0.65**	**0.70**	**1.16**	**0.646**
**Olive rockfish**	**0.04**	**0.05**	**0.26**	**0.37**	**0.82**	**0.732**
**Vermilion rockfish**	**0.04**	**0.03**	**0.25**	**0.37**	**0.73**	**0.807**
Lingcod	0.04	0.04	0.57	1.76	0.69	0.880
**Copper rockfish**	**0.02**	**0.02**	**0.12**	**0.20**	**0.35**	**0.919**
Kelp rockfish	0.008	0.008	0.08	0.20	0.08	0.934
**Brown rockfish**	**0.008**	**0.011**	**0.04**	**0.07**	**0.08**	**0.948**
**China rockfish**	**0.005**	**0.005**	**0.04**	**0.06**	**0.06**	**0.957**
Canary rockfish	0.005	0.005	0.06	0.07	0.03	0.966
Yellowtail rockfish	0.004	0.004	0.06	0.15	0.06	0.974
Cabezon	0.004	0.004	0.04	0.07	0.04	0.981
Kelp greenling	0.002	0.002	0.02	0.03	0.02	0.984
**Treefish**	**0.002**	**0.003**	**0.008**	**0.011**	**0.016**	**0.987**
**Black and yellow rockfish**	**0.001**	**0.002**	**0.008**	**0.011**	**0.013**	**0.990**
**Ocean whitefish**	**0.001**	**0.002**	**0.003**	**0.002**	**0.019**	**0.992**
**Bocaccio**	**0.001**	**0.003**	**0.001**	**0.001**	**0.020**	**0.994**
**Rosy rockfish**	**0.001**	**0.001**	**0.005**	**0.006**	**0.010**	**0.996**
**Starry rockfish**	**0.001**	**0.001**	**0.004**	**0.002**	**0.006**	**0.997**

Bolded species peaked in abundance following the MHW.

### Functional diversity

In addition to changes in species composition, we also observed a shift in trophic structure (functional diversity) in relation to the MHW (PERMANOVA: F_2,107_ = 29.62, R^2^ = 0.31, p < 0.001). Prior to the MHW, approximately 23% of the community consisted of planktivores, 53% mixed carnivores (piscivore/invertivore) and 21% piscivores both inside and outside the MPAs. Following the MHW, the fish community was dominated by planktivores comprising almost 60% of the entire fish community—driven primarily by increases in Blue/Deacon rockfish, while piscivores declined and made up < 5% of the fish community (Fig. 3b). Interestingly, the proportion of piscivores and mixed carnivores (combined) declined 30% inside MPAs and 43% in areas open to fishing following the MHW concomitant with increases in planktivores inside and outside reserves (Supplementary Fig. S3).

### Species-specific rates of change inside and outside MPAs

**Figure 5:**
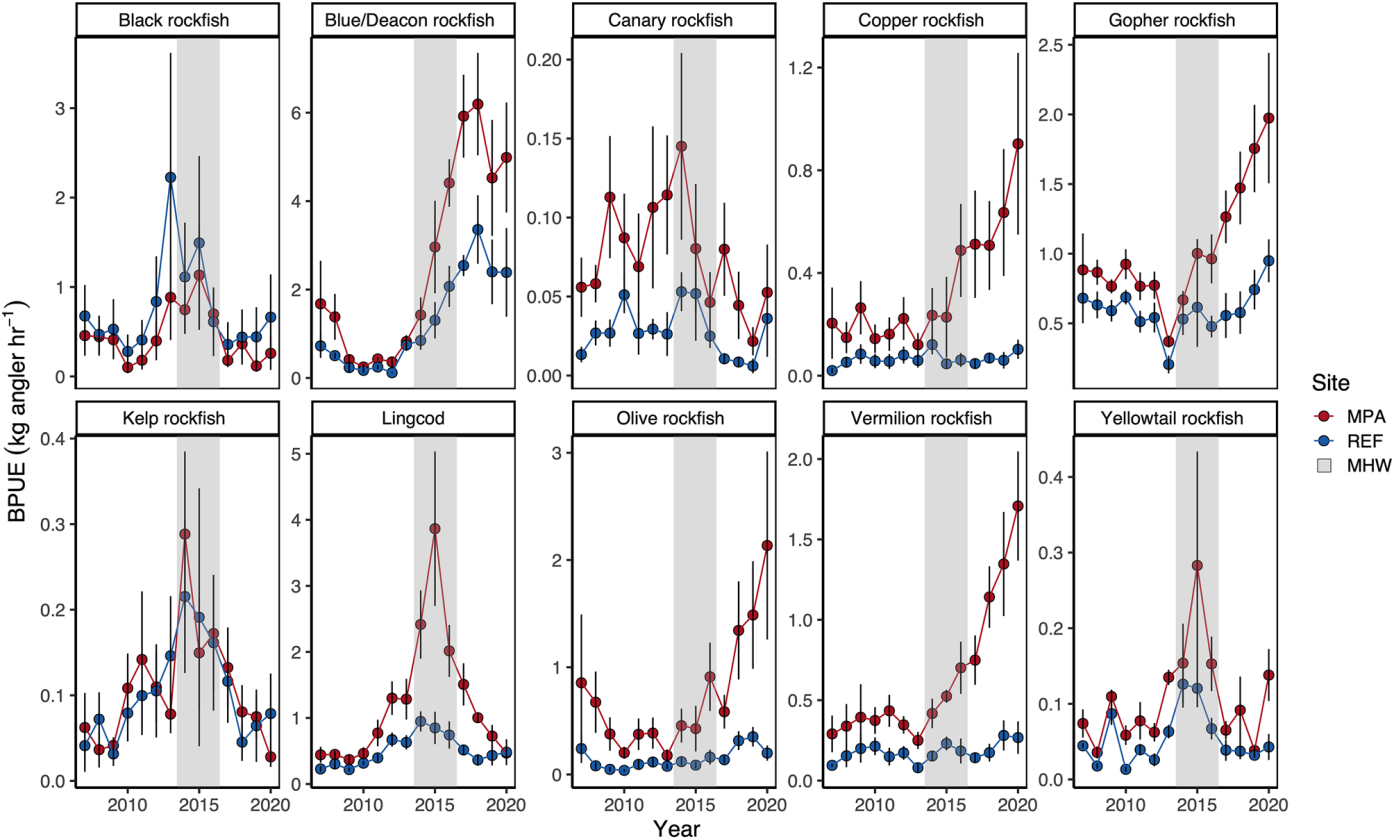
Trends in biomass per unit effort (BPUE) of the top ten most abundant fish species at MPA and reference (REF) sites before (2007–2013), during (2014–2016), and after (2017–2020) the marine heatwave (MHW). Points are annual means +/− 95% CI.

We observed that four species (Gopher rockfish, Copper rockfish [*Sebastes caurinus*], Olive rockfish and Vermilion rockfish [*Sebastes miniatus*]) increased faster in biomass inside MPAs compared to reference sites following the MHW, compared to the period prior to the heatwave (Fig. 5; Table 2). We also found that Lingcod (*Ophiodon elongatus*), Yellowtail rockfish (*Sebates flavidus*), and Kelp rockfish (*Sebastes atrovirens*) biomasses exhibited negative slopes, indicating declines in biomass in both the MPAs and reference sites following the MHW. Lingcod, in particular had much larger declines in biomass (~ 4 × greater) inside the MPAs compared to areas open to fishing (Table 2), because the biomass was much higher inside the MPAs prior to and during the heatwave. The Blue/Deacon rockfish complex also exhibited declines in biomass the MPAs after the heatwave, but there was little change in reference sites (Table 2). However, the Blue/Deacon rockfish complex had the highest overall biomass per unit effort (BPUE) compared to all other species, and the apparent decline stems from a peak in biomass in 2017 followed by relative decreases in biomass in more recent years inside the MPAs (Fig. 5; Supplementary Table S2). Although we determined there were differences in the rate of change in species-specific biomass, there were no statistical differences in the overall distribution of positive and negative slopes for all species before and after the MHW, in either MPAs (D = 0.24, p = 0.6) or the associated reference sites (D = 0.33, p = 0.19; Supplementary Fig. S4).

**Table 2:** The rate of change in biomass (biomass per unit effort; BPUE) of the 12 species most commonly caught during surveys along the Central coast region for MPA and Reference (REF) sites following the MHW.

Common name	Slope	
	MPA	REF
Black rockfish	− 0.09	0.02
Blue/Deacon rockfish complex	− 0.02	0.05
Brown rockfish	0.013	0.009
Canary rockfish	− 0.005	0.002
Copper rockfish	0.10	0.01
Gopher rockfish	0.25	0.11
Kelp rockfish	− 0.03	− 0.02
Lingcod	− 0.39	− 0.06
Olive rockfish	0.34	0.03
Rosy rockfish	0.002	0.002
Vermilion rockfish	0.26	0.03
Yellowtail rockfish	− 0.006	− 0.006

Negative values indicate a decline in biomass, positive values indicate an increase in biomass, and values close to zero indicate no change.

## Discussion

In this study, we investigated the effects of the 2014–2016 MHW in the California Current Ecosystem (CCE) on targeted nearshore fish communities inside and outside no-take MPAs using 14 years of hook-and-line data collected by CCFRP. Overall, we found that MPAs did not fully resist the effects of the heatwave, which depressed diversity and evenness in both MPAs and areas open to fishing and resulted in a shift in fish community composition and trophic structure. However, MPAs appeared to be more resilient to the heatwave; we define resilience as the ability of a system to resist changing and/or recover from a perturbation^39,40^. Specifically, we observed that marine reserves were more resistant to functional changes and taxonomic diversity recovered more rapidly from the MHW than in references areas open to fishing. Further, our results indicate that not all species responded similarly to the heatwave, with the biomass of specific species (e.g. Copper rockfish, Gopher rockfish, Vermilion rockfish) and trophic groups (e.g. planktivores) increasing dramatically following the heatwave, while others declined in conjunction with the temperature anomaly (e.g. Lingcod).

Shannon-Weiner diversity in both MPAs and areas open to fishing declined dramatically during the MHW on the central coast (47 and 40%, respectively). Surprisingly, the reduction in Shannon-Weiner diversity was greater inside MPAs; this may have resulted in part from the fact that Shannon-Weiner diversity was higher inside MPAs before the MHW and therefore had more scope to decline as hypothesized by the protection paradox^33^. Additionally, with the loss of Shannon-Weiner diversity there was a clear shift in community composition toward planktivorous species (e.g. the Blue/Deacon rockfish complex) and increased biomass of certain warm-water affinity species (e.g. Ocean whitefish)^4^. Previous studies reported that fish movements in response to the MHW varied geographically with fishes in some ecoregions moving poleward while others shifted to deeper depths^21,41^. For example, Cavole et al.^21^ found that highly mobile Yellowfin tuna (*Thunnus albacares*) were sited almost 10º N of their typical northernmost distribution during the MHW event, while there was no change in the poleward distribution of Alaskan pollack (*Gadus chalcogrammus*).

The declines in Shannon-Weiner diversity, evenness, and compositional shifts that we observed mirror those reported by Freedman et al.^32^ where fish communities both inside and outside of MPAs in the Channel Islands in southern California responded similarly to the heatwave, although those changes were mainly driven by increases in the abundance of non-targeted species with warm-water affinities, without commensurate declines in the cold-water affinity species. Southern and central California experience different oceanographic regimes, split by a major biogeographic break at Point Conception, and are characterized by different fish communities with different thermal affinities^42–44^. We observed similar responses in central California, which points to the broad spatial extent of the impacts of the 2014–2016 heatwave in the CCE. Globally marine heatwaves have drastically affected community structure in biodiversity hotspots in unique ways^16^. For example, in the Mediterranean Sea, a 2003 MHW caused mass mortality of more than 25 benthic macroinvertebrates, resulting in a loss of overall biodiversity^45^, while in 2011, a MHW in western Australia caused dramatic declines in canopy forming species and increases in warm-water species, shifting community structure from temperate towards more tropical assemblages^16,46^.

Theoretical predictions suggest that MPAs will enhance ecosystem resilience to disturbance through a variety of potential mechanisms, including increased population sizes and increased diversity^33,47^. MPAs also may improve critical biogenic habitat through trophic effects^27,29^. While not statistically significant (possibly due to a low number of years in the analysis [n = 4]), we found Shannon-Weiner diversity trended toward more rapid recovery inside the MPAs compared to the areas open to fishing in the years immediately following the heatwave (2017–2020). Specifically, we found that Shannon-Weiner diversity inside MPAs recovered 75% faster than adjacent reference sites in the 4 years following the MHW, suggesting that the MPAs are more resilient to MHWs in terms of taxonomic diversity. We also observed that functional diversity (trophic structure) was influenced by the MHW. Overall, there was a large decrease in proportional biomass of higher trophic level species such as piscivores and mixed carnivores in both MPAs and areas open to fishing. However, the proportional decline was dampened inside the MPA with only a 30% decline compared to 43% decline in areas open to fishing driven by a smaller loss of piscivores and an increase in the biomass of mixed carnivores inside the MPAs. This further suggests that MPAs may be more resistant to climatic disturbance events by maintaining higher functional diversity.

It is possible that the absence of fishing pressure inside MPAs allowed higher trophic level species to persist and taxonomic diversity to recover more quickly, highlighting how harvest may influence species abundance patterns and trophic interactions. However, it is important to note that neither the environment nor fish community has returned to pre-heatwave status during the timeframe of our study. Continued warmer water conditions and higher maximum water temperatures in conjunction with the protection of fishes inside MPAs may actually facilitate the establishment of new species in the community. For example, transitioning and adult male California sheephead, *Semicossyphus pulcher* which are uncommon north of Point Conception^48^, have been caught more often by CCFRP inside the central coast MPAs in recent years. This suggests MPAs may act as a refuge for fishery targeted species that are shifting northward with warming conditions, in contrast to previous findings that MPAs may resist colonization by tropical^49^ or non-native species^29,50^. In some cases, MPAs acting as a refuge for new species may be the desired result of a marine reserve. For instance, Papahānaumokuākea Marine National Monument in Hawaii was specifically designed as a climate refuge for coral reef communities^51^. However, the introduction and establishment of new species in a reserve may have cascading effects throughout the ecosystem and implications for the recovery of MPAs from anomalous climatic events into the future. For example, a 400% increase in herbivorous grazers on temperate reefs in Western Australia due to a MHW has led substantial declines in seaweed canopies and a regime shift to turf algae-dominated systems^7,52^.

Prior to the MHW, the fish community was dominated by higher trophic level species such as piscivores and mixed carnivores. However, during and following the 2014–2016 MHW, the fish communities inside and outside MPAs shifted towards the dominance of planktivorous species. Other studies reported increases in primary producers along the CCE during the MHW, many associated with harmful algal blooms^53^, and similar increases in other planktivorous species, perhaps due to life history or functional traits that allowed planktivorous species to thrive in warmer highly productive waters^54^. For example, there were record high numbers of larval northern anchovy and Pacific sardines captured off the coast of northern California and Oregon during the heatwave^55^. Biomass transfer to higher trophic levels and mean trophic level are expected to decrease under warming conditions due to faster and less efficient trophic transfer in marine ecosystems^56^ and has may result in collapses of mid-level consumers and top predators^57^. Food webs composed of a wide array of consumers across multiple trophic levels tend to increase ecosystem stability compared to communities dominated by few predators or lower trophic levels^58^. The influence of the MHW on higher order consumers appears to mimic the removal of top predators via fishing and may negate the positive effects of MPAs on biodiversity. Further, the reduction of predators and subsequent decrease in mean trophic level in the assemblage may have unexpected cascading effects, reducing ecosystem stability, and the ability of the marine communities to respond to future disturbance events^59^.

Strong upwelling drives the high productivity observed in the CCE during ‘normal’ years. During the MHW, the traditional upwelling cycle was altered causing higher NPP in the years following the heatwave^60^, potentially affecting the timing of the spring phytoplankton bloom and subsequent zooplankton or micronekton availability for fishes that consume species lower on the food web^61^. Higher than normal NPP and shifts in the spring bloom may have provided the conditions needed for the rapid proliferation of planktivory and a phenological mismatch for the larvae of non-planktivorous fishes in the CCE^3^. As these rapid changes in oceanography affect the larval and juvenile stages of marine fishes, the full effects of the heatwave may not be apparent in the fishery until years after an anomalous event. We found that the declines in diversity and evenness were strongly correlated with a 2-year lag in the Multivariate Ocean Climate Indicator (MOCI) and appear to be primarily driven by the rise in dominance of the Blue/Deacon rockfish complex during the MHW. The Blue/Deacon rockfish complex experienced strong recruitment in numerous years surrounding the MHW, possibly due to favorable oceanographic conditions^62,63^. Larvae of these species are released in the early spring^64^, which may have overlapped with the earlier spring bloom during the heatwave years. This latent effect of the heatwave paired with a successful recruitment event became apparent in the fishery 2–3 years after the onset of the heatwave in 2017 and 2018, where the Blue/Deacon rockfish complex became the most dominant group on the central coast in both biomass and abundance. Since our program samples fish with hook-and-line gear, generally most species will be a minimum of 2 years old before attaining a body size large enough to interact with the fishing gear. In addition, we found that many common species including Lingcod, Canary rockfish and Yellowtail rockfish peaked in biomass in 2014–2015 and then declined rapidly. In some cases, these changes in species-level biomass are possibly due to high recruitment during the cool and productive La Niña years^65^ preceding the MHW, followed by unsuccessful recruitment during the heatwave^66^. Additionally, as a result of persistent high water temperatures adult fishes may have perished or migrated to deeper cooler waters to find a climate refuge. The combination of fish movements and the booms and bust in recruitment of certain species directly contributed to the observed shifts in species composition and diversity following the 2014–2016 heatwave event. As many of these fishes are long-lived, successful recruitment events occur relatively infrequently and are strongly correlated with oceanographic processes^67^. Therefore, increased variability in oceanographic conditions that are predicted with climate change—especially including climatic anomalies such as heatwaves—may result in long-term impacts on the composition and productivity of coastal fisheries into the future.

Overall, our study supports previous findings that MHWs cause unexpected shifts in fish community composition and biodiversity in marine reserves and reference sites open to fishing^14,68^. We found that the Blue and Deacon rockfish complex dominated the rocky reef fish community following the MHW in both MPAs and areas open to fishing. Also, there was a clear shift in trophic diversity with a large proportional increase in planktivorous species in MPAs and areas open to fishing following the MHW. However, the decline in higher trophic level species was buffered inside the MPAs suggesting that communities (in terms of functional diversity) inside marine reserves may be more resistant to climatic anomalies. Lastly, we found that while no-take MPAs did not mitigate the immediate effects of anomalous warm temperatures on fisheries species in central California (similar to Freedman et al.^32^ from southern California), taxonomic diversity (e.g. Shannon-Weiner) in MPAs appeared to recover more rapidly than areas open to fishing in the four years immediately following the MHW. These results suggest a re-evaluation of expectations in how fish communities inside MPAs will respond to future climate change is needed, as the forces driving distributional shifts and species turnover occur at broader spatial scales. Finally, our study highlights the importance of long-term monitoring programs as critical tools to understand and predict past, present, and future changes in ecosystem structure and function under novel environmental and biological regimes.

## Methods

### CCFRP field sampling

CCFRP was initiated in 2007 as MPAs were established along the central coast of California as a collaboration with recreational anglers and commercial passenger fishing vessels (CPFVs) to survey targeted fish communities inside and outside of MPAs using a standardized, repeatable, scientifically rigorous methodology^38^. With the assistance of volunteer anglers we sampled four no-take marine reserves and associated reference sites open to fishing along the central coast of California (Año Nuevo State Marine Reserve (SMR), Point Lobos SMR, Piedras Blancas SMR and Point Buchon SMR; Fig. 1) annually between from 2007 to 2021. Reference sites were selected due proximity to the MPA (< = 10 km) and consisted of similar bathymetry, habitat, and oceanographic conditions. At each site (MPA or reference), 500 m by 500 m fixed grid cells were established in rocky reef habitat < 40 m in depth. The number of fixed grid cells delineated at each site varied based on the amount of suitable habitat in the designated depth range (22 at Año Nuevo, 17 at Point Lobos, 57 at Piedras Blancas and 22 at Point Buchon across both MPA and reference sites). For each sampling day (n = 6 per year), we randomly selected four 500 × 500 m sampling grid cells at each site (n = 12 for each MPA or reference site per year)^69,70^. Within each grid cell, CPFV captains selected three suitable locations to conduct 15-min fishing drifts. During each drift, volunteer anglers actively fished using a standardized set of hook and line fishing gear to capture a variety of species. During each drift, the number of anglers, total time spent fishing, location (latitude and longitude), depth, and habitat relief were recorded. For all fish captured, we identified each to species level, measured total length in cm, and then released them using a descending device (if needed) to minimize the effects of barotrauma^71^. For more details on sampling design see Starr et al.^69^. CCFRP sampling data are publicly available at the California Ocean Protection Council Data Repository^72^.

### Response variables

To standardize our sampling effort, we calculated both catch per unit effort (CPUE) and biomass per unit effort (BPUE) for grid cells in each MPA and reference site. CPUE was calculated as the total number of fish caught per species divided by the number of angler hours fished (no. fish * angler h^−1^) in each grid cell. We calculated biomass of each individual using published length–weight relationships for each species (Love et al. 1990; Froese and Pauly 2021), and then calculated BPUE as the total weight of fish caught in kilograms divided by the number of angler hours fished (kg * angler h^−1^).

We calculated species richness, Pielou’s evenness, and the Shannon–Wiener diversity index using our CPUE (i.e. relative abundance) values to determine changes in diversity across sites and through time. Species richness is the total number of unique species in a given area. Shannon–Wiener diversity combines species richness and their relative abundances, calculated as

$${H}^{^{\prime}}= -\sum_{i=1}^{R}{p}_{i}\,\mathrm{ ln}\,{ p}_{i},$$

where p_i_ is the proportion of species *i* for R number of species present in the sample. Pielou’s evenness is the proportion of the relative abundance of each species in an area between 0 and 1 and is calculated as

$${J}^{^{\prime}}=\frac{{H}^{^{\prime}}}{\mathrm{ln}(S)},$$

where Hʹ is Shannon–Wiener diversity and S is the total number of species in a sample, across all samples in the dataset.

### Environmental data extraction

We extracted a variety of environmental data metrics from public data repositories to examine the broad scale changes in oceanographic conditions in relation to the marine heatwave. We first extracted water temperature anomalies data for visualization from the NOAA ERDDAP Multi-scale Ultra-high Resolution (MUR) SST Analysis Anomaly dataset (https://coastwatch.pfeg.noaa.gov/erddap/index.html). These data were extracted as monthly means for the month of September for all sampling years (2007–2020). Sea Surface Temperature data (°C; SST) were collected from the Advanced Very High-Resolution Radiometer instrument aboard NOAA’s Polar Operational Environmental Satellites (Kilpatrick et al. 2001). SST measurements were collected daily from 2004 to 2020 at a 1.47 km spatial resolution, accurate to ± 0.7 °C. In addition to temperature, we examined net primary production (NPP), wind speed, and wave metrics (orbital velocity and significant wave height). These metrics are directly related to both upwelling and fishing ability through time influencing fish community composition and can be altered by climatic patterns such as the El Niño-Southern Oscillation (ENSO). NPP data (mg C m^−2^ day^−1^; NPP) were collected by the California Current Merged Satellite daily from 1996 to 2020 at a 4 km spatial resolution. Wind speed (m s^−1^) was extracted from the Coupled Ocean/Atmosphere Mesoscale Prediction System (COAMPS) with a 4-km resolution. Significant wave height (m) and wave orbital velocity (m s^−1^; derived from wave height and dominant period) were extracted from the Coastal Data Information Program, Integrative Oceanography Division, operated by the Scripps Institution of Oceanography spectral files at the station nearest each MPA. For all forementioned environmental variables, we extracted the mean monthly data for each CCFRP grid cell for the time period during sampling between the months of July and October from 2007 to 2020.

We also extracted the Multivariate Ocean Climate Indicator (MOCI) as a measure of multiple oceanographic conditions. MOCI combines a variety of local and regional oceanographic parameters including seasonal averages for upwelling index, sea level, alongshore wind, sea surface temperature, air temperature, sea level pressure, the Multivariate ENSO index (MEI), the Pacific Decadal Oscillation (PDO), and the Northern Oscillation Index (NOI) into a single value for every three-month period^73^. For subsequent analyses we used the July, August, and September MOCI value for each year.

### Statistical analyses

We first visualized sea surface temperature anomalies during the MHW compared to all sampling years across the Central coast to ensure our sampling sites were exposed to anomalous conditions during the MHW (Fig. 1; Supplementary Fig. S1). We then statistically assessed changes in broad-scale environmental conditions over time along the central coast using our four broad geographic locations (MPA and reference sites combined). From examination of changing oceanographic conditions, data were binned into three time periods: before (2007–2013), during (2014–2016) and after (2017–2020) the heatwave. To assess the differences in oceanographic conditions among MPAs and Reference sites in relation to the MHW, we conducted a two-way analysis of variance (ANOVA) for each environmental variable (mean SST, maximum SST, NPP, wind speed and wave height) by geographic location and time period (before, during and after the MHW). All analyses we conducted in R version 4.0.5^74^.

We then explored the differences in trajectories of diversity metrics (species richness, Pielou’s evenness, and Shannon-Weiner diversity) in relation to the MHW (before, during, and after). To evaluate if there were clear differences in diversity metrics among the grouped time periods both inside and outside MPAs (MPA vs. reference site), we conducted interactive two-way ANOVAs for each diversity metric and used a Tukey’s Post-hoc test for pairwise comparisons of diversity differences across time periods and among MPA status. To examine differences in diversity metrics (species richness, Shannon–Wiener diversity and Pielou’s evenness) through time between MPA and reference sites in relation to the MHW, we ran serial ANCOVAs for each time period, independently (dropping interaction terms when p > 0.2). We also assessed how regional oceanographic conditions associated with the MHW affected diversity metrics among MPAs and reference sites, using general linear models testing MOCI values with 1-year, 2-year, and 3-year temporal lags.

To assess changes in community structure inside MPAs and in areas open to fishing (reference sites) in relation to the 2014–2016 MHW, we ran a permutational analysis of variance (PERMANOVA) for both species composition and trophic structure (e.g. herbivore, planktivore, invertivore, mixed carnivore, and piscivore)^37^. To visualize the dissimilarity among communities in relation to the heatwave and among MPAs and reference sites, we conducted a non-metric multidimensional scaling (nMDS) ordination with a Bray–Curtis distribution. We calculated 95% confidence interval (CI) ellipses for each grouping and the corresponding vectors for species and environmental variables (SST, NPP, and wave metrics) that corresponded to differences in fish communities. To further assess which species contributed the most to the shifts in community structure in relation to the MHW and between MPA and reference sites, we conducted a Similarity Percentages (SIMPER) analysis. Due to the dominance of the Blue/Deacon rockfish complex (*Sebastes mystinus* and *Sebastes diaconus*) within our sampling, we also conducted all multivariate analyses described above with the Blue/Deacon rockfish complex removed to test for general patterns. All multivariate analyses were conducted with the ‘vegan’ package^75^.

To further assess which species were contributing to community shifts in relation to the MHW, we calculated the rate of change in biomass (BPUE) for 21 species regularly captured inside the MPAs and adjacent references sites in the time periods before, during and after the heatwave. Using the binned time periods, we ran a series of linear regressions for 21 species through time and extracted the slope of the regression lines using the ‘nlme’ package^76^. To determine if the distributions of slopes inside MPAs or reference sites varied before and after the MHW, we conducted a Kolmogorov–Smirnov (K–S) test. All methods reported are in accordance with ARRIVE guidelines.

### Ethical approval

All experiments were approved by San Jose State University Institutional Animal Care and Use Committee (IACUC protocols #824, #1021) to ensure all applicable institutional and/or national guidelines for the care and use of animals were followed. Collections of fishes were approved through a California Department of Fish and Wildlife Scientific Collecting Permit (#2613, S‐191210002‐19126‐001).

## Supplementary Information

Supplementary Information.

## Data Availability

CCFRP data that support the findings of this study are openly available in the California Ocean Protection Council DataOne Repository (Brooks et al. 2022) at https://opc.dataone.org/view/urn%3Auuid%3Af843f110-e691-4d26-bf12-3854a4b641cd. All other data and code used for these analyses are publicly available at https://github.com/slziegler/CCFRP-MHW.
