# Supplementary material for: Marine protected areas, marine heatwaves, and the resilience of nearshore fish communities
Source: Sci Rep. 2023 Jan 25;13:1405. doi: 10.1038/s41598-023-28507-1 (PMC9876911; doi:10.1038/s41598-023-28507-1)
Supplement: Supplementary file 1 — Supplementary Information. [file 41598_2023_28507_MOESM1_ESM.pdf]

Supplementary Material

Marine protected areas, marine heatwaves, and the resilience of nearshore fish communities.

Shelby L. Ziegler[1,2]*, Jasmin M. Johnson[3], Rachel O. Brooks[1], Erin M. Johnston[4], Jacklyn L. Mohay[1], Benjamin I. Ruttenberg[4], Richard M. Starr[1], Grant T. Waltz[4], Dean E. Wendt[4], & Scott L. Hamilton[1]

1. Moss Landing Marine Laboratories, San Jose State University, Moss Landing, CA 95039 USA

2. Odum School of Ecology, University of Georgia, Athens, GA 30602 USA

3. Department of Marine Science, California State University Monterey Bay, Seaside, CA 93955 USA

4. Center for Coastal Marine Sciences, Biological Sciences Department, California Polytechnic State University, San Luis Obispo, CA 93407 USA

* Corresponding author: Shelby L Ziegler, shelbylziegler@gmail.com

Table S1. Environmental data for all years sampled inside and outside MPAs (means +/- SE). Values for SST and Max SST in ºC, NPP in mg C m^{-2} day^{-1}, Wave orbital velocity in m s^{-1}, significant wave height in m, and wind speed in m s^{-1}.

Year	MOCI	Area	Site	SST	Max SST	NPP	Orbital velocity	Wave height	Wind speed
			MPA	13.6+/-0.16	16.82+/-0.35	2709.2+/-100.77	277.15+/-30.07	1.52+/-0.04	
		Año Nuevo	REF	13.49+/-0.32	14.12+/-0.35	2989.32+/-181.69	255.45+/-20.85	1.57+/-0.04	
			MPA	13.74+/-0.13	14.61+/-0.3	1058.2+/-56.46	257.71+/-26.76	1.55+/-0.04	
		Point Lobos	REF	13.83+/-0.14	14.64+/-0.32	1545.89+/-135.11	280.33+/-24.59	1.61+/-0.04	
			MPA	13.69+/-0.07	15.46+/-0.14	1473.09+/-41.02	266.46+/-14.72	1.53+/-0.02	
		Piedras Blancas	REF	13.72+/-0.11	15.66+/-0.26	1521.54+/-41.97	129.89+/-10.54	1.09+/-0.01	
			MPA	13.86+/-0.19	15.27+/-0.4	1522.09+/-51.79	282.37+/-26.28	1.59+/-0.04	
2007	-0.85	Point Buchon	REF	14.2+/-0.22	16.03+/-0.32	1797.05+/-103.96	283.04+/-20.86	1.63+/-0.03	
			MPA	13.58+/-0.17	22.85+/-0.5	2194.46+/-60.76	501.58+/-63.29	1.54+/-0.04	
		Año Nuevo	REF	12.79+/-0.26	19.18+/-0.58	2487.25+/-177.2	436.51+/-47.41	1.6+/-0.04	
			MPA	13.08+/-0.3	20.92+/-0.68	1265.74+/-145.73	409.34+/-55.77	1.55+/-0.04	
		Point Lobos	REF	13.44+/-0.23	19.67+/-1.77	1047.96+/-56.32	446.65+/-53.51	1.62+/-0.04	
			MPA	14.27+/-0.11	22.72+/-0.81	1535.54+/-35.69	425.76+/-29.61	1.57+/-0.02	
		Piedras Blancas	REF	13.89+/-0.09	25.44+/-0.79	1735.59+/-62.48	203.79+/-15.93	1.14+/-0.01	
			MPA	14.28+/-0.26	24.21+/-0.62	1894.49+/-176.74	387.5+/-42.96	1.59+/-0.04	
2008	0.68	Point Buchon	REF	14.55+/-0.08	21.09+/-0.48	2056.78+/-126.25	373.12+/-35.82	1.61+/-0.03	
			MPA	12.96+/-0.17	14.48+/-0.17	2384.03+/-98.46	225.42+/-12.49	1.47+/-0.04	
		Año Nuevo	REF	13.34+/-0.38	14.84+/-0.25	2317.85+/-218.99	211.65+/-11.16	1.51+/-0.03	
			MPA	12.95+/-0.45	14.28+/-0.18	975.08+/-56.55	219.43+/-15.08	1.47+/-0.04	
		Point Lobos	REF	13.42+/-0.25	14.78+/-0.47	998.42+/-92.42	229.2+/-13.39	1.51+/-0.03	
			MPA	13.59+/-0.05	15.41+/-0.09	2363.81+/-90.52	265.08+/-10.47	1.58+/-0.02	
		Piedras Blancas	REF	13.68+/-0.05	15.73+/-0.05	2443.76+/-87.08	114.48+/-3.95	1.13+/-0.01	
			MPA	14.09+/-0.15	15.17+/-0.21	2070.8+/-204.63	240.65+/-19.03	1.54+/-0.04	
2009	-1.47	Point Buchon	REF	14.25+/-0.11	16.22+/-0.2	2612.71+/-195.99	252.27+/-17.05	1.58+/-0.03	
			MPA	13.06+/-0.06	14.57+/-0.14	2539.7+/-138.02	365.96+/-29.1	1.71+/-0.05	
		Año Nuevo	REF	13.02+/-0.13	14.67+/-0.13	3028.36+/-235.55	337.35+/-25.51	1.73+/-0.05	
			MPA	13.13+/-0.08	14.25+/-0.12	1342.83+/-182.54	326.17+/-30.79	1.68+/-0.06	
		Point Lobos	REF	13.44+/-0.08	15.04+/-0.17	2217.08+/-240.78	359.72+/-28.85	1.74+/-0.05	
			MPA	13.25+/-0.06	15.11+/-0.07	2295.79+/-59.1	411.23+/-21.31	1.81+/-0.03	
		Piedras Blancas	REF	13.45+/-0.08	14.81+/-0.12	2161.57+/-56.79	153.15+/-7.4	1.3+/-0.02	
			MPA	14.31+/-0.26	16.77+/-0.07	1980.48+/-109.19	333.32+/-29.66	1.76+/-0.05	
2010	-4.62	Point Buchon	REF	13.63+/-0.15	16.49+/-0.26	2735.5+/-144.39	342.65+/-23.27	1.8+/-0.04	
			MPA	13.98+/-0.17	18.1+/-0.5	2096.6+/-79.25	199.86+/-10.9	1.44+/-0.03	
		Año Nuevo	REF	14.46+/-0.14	16.05+/-0.06	2810.39+/-282.98	199.73+/-11.42	1.48+/-0.03	
			MPA	14.4+/-0.2	16.14+/-0.07	2316.21+/-221.95	234.91+/-16.78	1.61+/-0.04	
		Point Lobos	REF	14.37+/-0.12	16.55+/-0.1	1398.02+/-93.36	267.58+/-19.43	1.65+/-0.03	
			MPA	14.27+/-0.07	16.66+/-0.03	2455.09+/-78.16	259.77+/-8.86	1.68+/-0.02	
		Piedras Blancas	REF	14.06+/-0.09	16.78+/-0.01	2453.06+/-89.53	114.5+/-3.61	1.21+/-0.01	
			MPA	14.72+/-0.05	16.16+/-0.15	2893.8+/-341.48	238.29+/-15.9	1.63+/-0.03	
2011	-2.04	Point Buchon	REF	15.03+/-0.06	16.31+/-0.03	3040.32+/-237.45	247.15+/-12.26	1.67+/-0.02	
			MPA	13.32+/-0.16	16.08+/-0.09	1896.84+/-121.19	297.03+/-20.26	1.52+/-0.04	
		Año Nuevo	REF	13.25+/-0.18	15.51+/-0.11	1893.97+/-131.79	270.54+/-17.29	1.53+/-0.03	
			MPA	13.45+/-0.17	15.4+/-0.11	2167.58+/-492.53	306.79+/-28.75	1.68+/-0.05	
		Point Lobos	REF	13.64+/-0.15	16.01+/-0.09	3327.79+/-466.79	326.23+/-25.86	1.72+/-0.04	
			MPA	14.07+/-0.08	17.05+/-0.09	1728.76+/-73.08	303.82+/-13.73	1.65+/-0.02	
		Piedras Blancas	REF	14.02+/-0.08	16.96+/-0.13	1970.25+/-98.18	130.97+/-6.42	1.15+/-0.01	
			MPA	14.91+/-0.13	16.82+/-0.06	2393.27+/-292.95	254.28+/-20.7	1.57+/-0.04	
2012	-3.93	Point Buchon	REF	14.78+/-0.11	16.95+/-0.06	2138.27+/-94.98	260.4+/-18.46	1.61+/-0.04	
			MPA	13.6+/-0.15	15.83+/-0.28	2889.61+/-123.75	177.5+/-7.91	1.33+/-0.03	11.63+/-0.28
		Año Nuevo	REF	13.76+/-0.23	15.94+/-0.36	3614.79+/-214.7	170.02+/-6.66	1.36+/-0.03	12.49+/-0.31
			MPA	13.92+/-0.33	16.25+/-0.02	1923.88+/-155.45	211.55+/-11.71	1.45+/-0.04	12.11+/-0.31
		Point Lobos	REF	14.21+/-0.27	15.81+/-0.12	3547.37+/-640.16	211.62+/-10.74	1.48+/-0.03	11.77+/-0.22
			MPA	14.19+/-0.07	20.32+/-0.43	2940.37+/-62.33	228.99+/-7.45	1.51+/-0.02	14.41+/-0.15
		Piedras Blancas	REF	13.83+/-0.05	18.07+/-0.36	3214.24+/-66.04	87.08+/-2.55	1.03+/-0.01	13.21+/-0.14
			MPA	14.87+/-0.11	19.71+/-0.39	2358.32+/-112.92	207.67+/-13.3	1.42+/-0.04	12.58+/-0.33
2013	-0.41	Point Buchon	REF	14.79+/-0.08	19.69+/-0.06	2789.13+/-103.29	212.12+/-9.25	1.46+/-0.03	12.57+/-0.35

			MPA	16.02+/-0.06	18.56+/-0.04	2243.48+/-114.14	243.12+/-14.77	1.48+/-0.03	11.5+/-0.25
		Año Nuevo	REF	15.64+/-0.08	18.39+/-0.23	2615.24+/-199.71	221.03+/-12.61	1.5+/-0.03	12.55+/-0.25
			MPA	16.73+/-0.12	18.49+/-0.23	1803.4+/-164.44	224.88+/-18.59	1.56+/-0.04	13.07+/-0.39
		Point Lobos	REF	16.19+/-0.16	18.05+/-0.07	2022.33+/-255.56	242.59+/-17.65	1.6+/-0.04	12.31+/-0.32
			MPA	16.75+/-0.06	18.96+/-0.03	2009.29+/-72.54	270.39+/-12.28	1.58+/-0.02	14.35+/-0.12
		Piedras Blancas	REF	16.59+/-0.07	19.26+/-0.05	1956.93+/-62.86	134.09+/-7.85	1.12+/-0.01	13.78+/-0.12
			MPA	17.46+/-0.21	23.49+/-0.64	1929.24+/-215.72	241.41+/-19.25	1.49+/-0.04	12.97+/-0.25
2014	8.47	Point Buchon	REF	17.29+/-0.14	20.62+/-0.01	1632.62+/-47.68	247.84+/-15.94	1.53+/-0.03	12.84+/-0.24
			MPA	15.27+/-0.06	18.2+/-0.06	2252.12+/-103.6	220.89+/-24.73	1.37+/-0.04	11.43+/-0.21
		Año Nuevo	REF	15.3+/-0.08	17.63+/-0.06	2421.96+/-175.38	210.08+/-20.88	1.4+/-0.04	12.42+/-0.24
			MPA	15.7+/-0.12	18.99+/-0.4	1600.39+/-96.7	241.22+/-28.45	1.51+/-0.05	12.18+/-0.29
		Point Lobos	REF	16.01+/-0.16	19.11+/-0.32	1945.28+/-255.34	246.71+/-25.02	1.55+/-0.04	11.51+/-0.25
			MPA	16.71+/-0.1	22.48+/-0.27	1722.72+/-55.62	257.34+/-17.86	1.53+/-0.02	14.4+/-0.1
		Piedras Blancas	REF	16.68+/-0.11	22.04+/-0.23	1605.99+/-51.94	100.66+/-4.91	1.1+/-0.01	13.44+/-0.09
			MPA	17.31+/-0.18	21.46+/-0.11	1632.03+/-123.4	228.35+/-29.62	1.46+/-0.04	12.73+/-0.19
2015	11.18	Point Buchon	REF	17.48+/-0.13	21.99+/-0.1	1826.47+/-107.19	235.81+/-26.27	1.49+/-0.04	12.95+/-0.2
			MPA	13.56+/-0.16	16.15+/-0.03	1988.54+/-111.03	322.95+/-23.25	1.78+/-0.06	12.87+/-0.25
		Año Nuevo	REF	14.26+/-0.18	16.63+/-0.13	2367.61+/-165.88	297.55+/-18.12	1.78+/-0.05	13.95+/-0.26
			MPA	14.24+/-0.3	19.88+/-0.06	1787.5+/-174.64	342.43+/-34.11	1.87+/-0.06	13.54+/-0.33
		Point Lobos	REF	14.42+/-0.27	19.35+/-0.44	2009.65+/-311.65	355.47+/-31.66	1.95+/-0.06	13.1+/-0.28
			MPA	14.29+/-0.08	19.46+/-0.74	1396.55+/-33.45	355.27+/-19.14	1.84+/-0.03	14.72+/-0.05
		Piedras Blancas	REF	14.08+/-0.06	16.45+/-0.03	1780.71+/-98.04	141.71+/-7.54	1.29+/-0.02	13.91+/-0.06
			MPA	14.54+/-0.12	16.96+/-0.15	1872.66+/-144.34	291.05+/-25.44	1.73+/-0.05	13.24+/-0.18
2016	-0.77	Point Buchon	REF	14.77+/-0.07	16.74+/-0.12	1970.94+/-50.83	299.37+/-24.33	1.76+/-0.05	13.33+/-0.18
			MPA	13.98+/-0.24	20.12+/-0.62	2672.17+/-110.72	350.14+/-49.31	1.49+/-0.04	11.72+/-0.24
		Año Nuevo	REF	13.83+/-0.25	19.4+/-0.35	2742.02+/-209.42	305.92+/-31.41	1.52+/-0.03	12.47+/-0.31
			MPA	13.87+/-0.22	22.57+/-1.91	2048.16+/-191.35	374.9+/-50.87	1.62+/-0.05	12.19+/-0.41
		Point Lobos	REF	14.08+/-0.27	20.29+/-1.22	1835.68+/-111.63	388.67+/-48.42	1.68+/-0.04	11.39+/-0.36
			MPA	14.75+/-0.07	25.61+/-0.67	2395.04+/-61.5	360.72+/-27.76	1.56+/-0.02	13.88+/-0.11
		Piedras Blancas	REF	14.64+/-0.06	22.99+/-0.9	2807.19+/-86.96	134.84+/-8.97	1.13+/-0.02	13.34+/-0.12
			MPA	15.38+/-0.13	20.65+/-1.13	2617.84+/-168.69	292.71+/-31.7	1.48+/-0.04	12.47+/-0.3
2017	3.65	Point Buchon	REF	15.47+/-0.12	17.87+/-0.01	2884.05+/-185.22	303.74+/-29.73	1.5+/-0.03	12.38+/-0.28
			MPA	14.06+/-0.12	20.62+/-0.7	2308.44+/-58.9	298.85+/-31.41	1.42+/-0.04	11.62+/-0.16
		Año Nuevo	REF	13.39+/-0.31	19.88+/-0.18	2554.54+/-174.29	277.97+/-27.2	1.48+/-0.03	12.14+/-0.2
			MPA	14.54+/-0.18	18.38+/-0.3	1405.61+/-107.14	297.96+/-33.65	1.64+/-0.04	11.82+/-0.27
		Point Lobos	REF	14.65+/-0.18	17.99+/-0.16	1508.8+/-133.55	321.28+/-34.28	1.68+/-0.04	11.57+/-0.24
			MPA	15.15+/-0.07	23.91+/-0.73	1783.86+/-31.86	285.93+/-18.53	1.51+/-0.02	13.95+/-0.12
		Piedras Blancas	REF	15.1+/-0.06	24.77+/-0.97	2285.77+/-88.03	111.79+/-5.87	1.07+/-0.01	13.57+/-0.12
			MPA	15.98+/-0.18	21.9+/-0.78	2532.08+/-217.87	249.3+/-25.34	1.43+/-0.04	13.29+/-0.14
2018	0.44	Point Buchon	REF	15.91+/-0.11	19.9+/-0.23	2271.17+/-72.22	260.17+/-23.92	1.45+/-0.03	13.34+/-0.14
			MPA	13.33+/-0.11	20.05+/-1.21	2862.93+/-132.24	399.19+/-45.79	1.56+/-0.04	12.15+/-0.24
		Año Nuevo	REF	13.75+/-0.13	18.89+/-1.52	3611.92+/-207.13	343.49+/-31.6	1.58+/-0.04	12.89+/-0.32
			MPA	13.71+/-0.13	18.07+/-0.48	1589.42+/-97.22	313.54+/-30.92	1.63+/-0.04	13.79+/-0.56
		Point Lobos	REF	13.92+/-0.14	24.95+/-1.92	2370.36+/-213.06	328.26+/-26.77	1.69+/-0.04	13.41+/-0.45
			MPA	14.15+/-0.04	24.08+/-0.23	2762.4+/-46.55	338.88+/-20.16	1.61+/-0.02	14.82+/-0.08
		Piedras Blancas	REF	13.91+/-0.06	19.34+/-0.83	3253.42+/-83.89	139.04+/-7.29	1.16+/-0.01	14.11+/-0.06
			MPA	14.47+/-0.09	22.52+/-2.13	2907.2+/-169.64	286.1+/-27.15	1.54+/-0.04	13.27+/-0.24
2019	1.5	Point Buchon	REF	14.78+/-0.07	20.88+/-1.73	3853.29+/-91.21	296.35+/-23.93	1.56+/-0.03	13.22+/-0.24
			MPA	14.96+/-0.52	19.76+/-1.06		205.09+/-16.13	1.45+/-0.04	12.11+/-0.19
		Año Nuevo	REF	15.13+/-0.36	23.42+/-1.34		195.15+/-13.92	1.5+/-0.04	12.47+/-0.2
			MPA	14.4+/-0.43	20.21+/-1.3		236.69+/-21.33	1.62+/-0.05	12.47+/-0.28
		Point Lobos	REF	14.39+/-0.35	18.4+/-1.11		243.44+/-19.31	1.64+/-0.05	12.45+/-0.23
			MPA	14.26+/-0.1	23.55+/-0.66		235.39+/-13.83	1.56+/-0.02	14.62+/-0.11
		Piedras Blancas	REF	14.34+/-0.1	17.74+/-0.1		84.32+/-3.27	1.08+/-0.01	13.86+/-0.13
			MPA	15.14+/-0.19	20.15+/-0.1		208.7+/-19.38	1.52+/-0.04	13.16+/-0.24
2020	0.59	Point Buchon	REF	15.82+/-0.45	21.55+/-0.65		219.63+/-15.26	1.56+/-0.04	13.16+/-0.25

Table S2. Mean BPUE +/- SE across all sampling years for species contributing to differences in community (from SIMPER) before, during, and after the MHW.

Species	Site	2007	2008	2009	2010	2011	2012	2013	2014	2015	2016	2017	2018	2019	2020
Black and Yellow Rockfish	MPA	0.006+/-0.003	0.008+/-0.004	0.008+/-0.004	0.017+/-0.009	0.013+/-0.008	0.001+/-0.001	0.001+/-0.001	0.007+/-0.003	0.016+/-0.01	0.004+/-0.002	0.006+/-0.003	0.002+/-0.002	0.004+/-0.002	0.027+/-0.017
	REF	0.011+/-0.009	0.007+/-0.003	0.009+/-0.005	0.005+/-0.002	0.009+/-0.006	0.014+/-0.006	0.003+/-0.002	0.005+/-0.003	0.029+/-0.014	0.004+/-0.003	0.021+/-0.006	0.016+/-0.007	0.004+/-0.003	0.021+/-0.011
Black Rockfish	MPA	0.366+/-0.098	0.473+/-0.077	0.385+/-0.108	0.101+/-0.04	0.186+/-0.057	0.405+/-0.089	0.896+/-0.184	0.751+/-0.145	0.917+/-0.216	0.699+/-0.132	0.165+/-0.045	0.352+/-0.108	0.124+/-0.044	0.264+/-0.092
	REF	0.657+/-0.098	0.53+/-0.085	0.536+/-0.111	0.268+/-0.066	0.414+/-0.084	0.849+/-0.185	2.288+/-0.479	1.108+/-0.199	1.152+/-0.32	0.62+/-0.126	0.38+/-0.09	0.449+/-0.12	0.443+/-0.129	0.663+/-0.197
Blue/Deacon Rockfish complex	MPA	1.872+/-0.359	1.397+/-0.184	0.415+/-0.082	0.248+/-0.034	0.43+/-0.062	0.363+/-0.07	0.824+/-0.125	1.402+/-0.198	2.821+/-0.429	4.41+/-0.425	5.972+/-0.54	6.281+/-0.873	4.516+/-0.632	4.951+/-0.68
	REF	0.689+/-0.103	0.481+/-0.057	0.238+/-0.045	0.168+/-0.038	0.244+/-0.056	0.12+/-0.024	0.749+/-0.111	0.874+/-0.181	1.223+/-0.233	2.053+/-0.286	2.572+/-0.342	3.297+/-0.442	2.4+/-0.355	2.387+/-0.464
Brown Rockfish	MPA	0.017+/-0.013	0.024+/-0.01	0.062+/-0.04	0.08+/-0.029	0.069+/-0.021	0.075+/-0.026	0.102+/-0.042	0.079+/-0.04	0.111+/-0.05	0.102+/-0.039	0.118+/-0.051	0.068+/-0.038	0.106+/-0.046	0.185+/-0.074
	REF	0.008+/-0.005	0.04+/-0.019	0.033+/-0.014	0.034+/-0.017	0.007+/-0.006	0.02+/-0.01	0.005+/-0.003	0.02+/-0.01	0.042+/-0.022	0.021+/-0.013	0.037+/-0.014	0.017+/-0.009	0.037+/-0.017	0.067+/-0.023
Cabezon	MPA	0.028+/-0.011	0.017+/-0.009	0.036+/-0.015	0.027+/-0.016	0.051+/-0.017	0.058+/-0.017	0.035+/-0.012	0.06+/-0.021	0.132+/-0.05	0.046+/-0.013	0.029+/-0.01	0.046+/-0.016	0.027+/-0.012	0.004+/-0.004
	REF	0.016+/-0.008	0.044+/-0.011	0.043+/-0.017	0.043+/-0.016	0.051+/-0.021	0.027+/-0.011	0.037+/-0.013	0.042+/-0.014	0.065+/-0.028	0.081+/-0.03	0.063+/-0.019	0.028+/-0.011	0.067+/-0.019	0.027+/-0.015
Calico Rockfish	MPA	0+/-0	0+/-0	0+/-0	0+/-0	0+/-0	0+/-0	0+/-0	0+/-0	0+/-0	0+/-0	0.001+/-0	0+/-0	0+/-0	0+/-0
	REF	0+/-0	0+/-0	0+/-0	0+/-0	0+/-0	0+/-0	0+/-0	0+/-0	0.001+/-0	0+/-0	0+/-0	0+/-0	0+/-0	0+/-0
Canary Rockfish	MPA	0.048+/-0.021	0.056+/-0.015	0.112+/-0.032	0.087+/-0.023	0.07+/-0.023	0.108+/-0.036	0.115+/-0.027	0.147+/-0.047	0.072+/-0.039	0.046+/-0.015	0.075+/-0.022	0.043+/-0.02	0.023+/-0.009	0.054+/-0.022
	REF	0.013+/-0.005	0.023+/-0.007	0.027+/-0.012	0.051+/-0.014	0.027+/-0.01	0.03+/-0.01	0.027+/-0.01	0.052+/-0.011	0.047+/-0.024	0.025+/-0.007	0.011+/-0.004	0.008+/-0.005	0.006+/-0.003	0.036+/-0.015
China Rockfish	MPA	0.074+/-0.019	0.044+/-0.008	0.061+/-0.014	0.059+/-0.013	0.041+/-0.01	0.057+/-0.011	0.032+/-0.009	0.058+/-0.013	0.078+/-0.02	0.056+/-0.012	0.053+/-0.014	0.034+/-0.011	0.057+/-0.015	0.065+/-0.019
	REF	0.04+/-0.009	0.037+/-0.011	0.041+/-0.012	0.052+/-0.015	0.025+/-0.006	0.062+/-0.015	0.018+/-0.005	0.043+/-0.011	0.053+/-0.017	0.039+/-0.01	0.056+/-0.019	0.047+/-0.015	0.042+/-0.01	0.093+/-0.033
Copper Rockfish	MPA	0.228+/-0.052	0.139+/-0.03	0.27+/-0.055	0.144+/-0.033	0.157+/-0.039	0.221+/-0.049	0.118+/-0.027	0.235+/-0.044	0.18+/-0.07	0.488+/-0.082	0.498+/-0.106	0.516+/-0.078	0.606+/-0.118	0.88+/-0.169
	REF	0.019+/-0.007	0.041+/-0.012	0.082+/-0.024	0.058+/-0.017	0.054+/-0.013	0.079+/-0.02	0.056+/-0.015	0.121+/-0.019	0.038+/-0.017	0.06+/-0.016	0.046+/-0.015	0.067+/-0.016	0.06+/-0.02	0.103+/-0.029
Gopher Rockfish	MPA	0.99+/-0.092	0.87+/-0.063	0.769+/-0.056	0.924+/-0.066	0.764+/-0.065	0.772+/-0.06	0.371+/-0.039	0.669+/-0.052	0.995+/-0.089	0.963+/-0.078	1.261+/-0.103	1.485+/-0.141	1.759+/-0.164	1.996+/-0.206
	REF	0.693+/-0.067	0.617+/-0.054	0.587+/-0.055	0.69+/-0.05	0.509+/-0.052	0.535+/-0.063	0.197+/-0.024	0.531+/-0.051	0.579+/-0.106	0.477+/-0.048	0.516+/-0.054	0.579+/-0.079	0.742+/-0.077	0.949+/-0.092
Kelp Greenling	MPA	0.023+/-0.006	0.024+/-0.006	0.028+/-0.008	0.034+/-0.008	0.031+/-0.009	0.024+/-0.006	0.012+/-0.004	0.026+/-0.007	0.041+/-0.012	0.035+/-0.008	0.019+/-0.006	0.017+/-0.007	0.02+/-0.005	0.018+/-0.009
	REF	0.041+/-0.011	0.016+/-0.004	0.019+/-0.006	0.022+/-0.006	0.028+/-0.007	0.031+/-0.007	0.009+/-0.004	0.021+/-0.005	0.042+/-0.009	0.03+/-0.007	0.031+/-0.01	0.013+/-0.005	0.004+/-0.002	0.012+/-0.006
Kelp Rockfish	MPA	0.072+/-0.019	0.037+/-0.009	0.043+/-0.01	0.108+/-0.033	0.138+/-0.042	0.109+/-0.025	0.078+/-0.018	0.285+/-0.061	0.156+/-0.04	0.172+/-0.041	0.141+/-0.036	0.082+/-0.02	0.072+/-0.02	0.028+/-0.009
	REF	0.038+/-0.013	0.057+/-0.014	0.031+/-0.011	0.08+/-0.017	0.097+/-0.026	0.102+/-0.027	0.138+/-0.038	0.22+/-0.047	0.147+/-0.054	0.157+/-0.048	0.108+/-0.024	0.044+/-0.013	0.064+/-0.026	0.079+/-0.031
Lingcod	MPA	0.475+/-0.107	0.456+/-0.054	0.365+/-0.064	0.462+/-0.059	0.784+/-0.121	1.315+/-0.136	1.299+/-0.172	2.394+/-0.255	3.224+/-0.466	2.016+/-0.222	1.434+/-0.177	0.998+/-0.134	0.741+/-0.117	0.466+/-0.089
	REF	0.234+/-0.048	0.324+/-0.059	0.22+/-0.043	0.316+/-0.043	0.396+/-0.051	0.674+/-0.078	0.636+/-0.105	0.956+/-0.144	0.701+/-0.16	0.732+/-0.099	0.524+/-0.085	0.365+/-0.076	0.432+/-0.09	0.481+/-0.119
Ocean Whitefish	MPA	0.006+/-0.006	0.008+/-0.006	0.022+/-0.013	0.006+/-0.006	0+/-0	0.005+/-0.005	0+/-0	0+/-0	0+/-0	0.013+/-0.01	0.005+/-0.005	0.03+/-0.02	0.024+/-0.021	0.06+/-0.033
	REF	0+/-0	0+/-0	0+/-0	0+/-0	0+/-0	0+/-0	0+/-0	0+/-0	0+/-0	0+/-0	0+/-0	0.002+/-0.002	0.008+/-0.008	0.016+/-0.011
Olive Rockfish	MPA	0.979+/-0.22	0.643+/-0.114	0.383+/-0.082	0.202+/-0.035	0.365+/-0.07	0.382+/-0.068	0.174+/-0.033	0.448+/-0.075	0.398+/-0.084	0.911+/-0.14	0.569+/-0.082	1.369+/-0.226	1.435+/-0.269	2.069+/-0.526
	REF	0.233+/-0.039	0.076+/-0.021	0.048+/-0.014	0.039+/-0.012	0.092+/-0.025	0.115+/-0.03	0.074+/-0.019	0.124+/-0.031	0.086+/-0.024	0.158+/-0.042	0.136+/-0.029	0.316+/-0.054	0.352+/-0.062	0.2+/-0.039
Pacific or Chub Mackerel	MPA	0+/-0	0+/-0	0+/-0	0+/-0	0+/-0	0+/-0	0+/-0	0+/-0	0.009+/-0.007	0+/-0	0+/-0	0.001+/-0.001	0.004+/-0.004	0.002+/-0.002
	REF	0+/-0	0+/-0	0+/-0	0+/-0	0+/-0	0+/-0	0+/-0	0+/-0	0.018+/-0.01	0.001+/-0.001	0.001+/-0.001	0.005+/-0.003	0.001+/-0.001	0.001+/-0.001
Rosy Rockfish	MPA	0.018+/-0.004	0.008+/-0.003	0.005+/-0.002	0.006+/-0.002	0.005+/-0.002	0.006+/-0.002	0.005+/-0.003	0.008+/-0.004	0.005+/-0.002	0.004+/-0.001	0.008+/-0.002	0.011+/-0.004	0.008+/-0.003	0.015+/-0.004
	REF	0.009+/-0.004	0.002+/-0.001	0.002+/-0.001	0.003+/-0.002	0.005+/-0.002	0.005+/-0.002	0.001+/-0.001	0.005+/-0.002	0.008+/-0.003	0.006+/-0.002	0.005+/-0.003	0.013+/-0.005	0.01+/-0.003	0.012+/-0.004
Starry Rockfish	MPA	0.018+/-0.006	0.003+/-0.002	0.006+/-0.003	0.003+/-0.002	0+/-0	0.004+/-0.002	0.001+/-0.001	0.002+/-0.002	0.002+/-0.002	0.001+/-0.001	0.003+/-0.002	0.016+/-0.008	0.007+/-0.004	0.014+/-0.007
	REF	0.005+/-0.003	0.002+/-0.001	0.006+/-0.003	0.002+/-0.002	0.004+/-0.002	0.005+/-0.003	0.004+/-0.003	0.003+/-0.002	0.002+/-0.002	0.002+/-0.001	0+/-0	0.002+/-0.002	0.003+/-0.003	0.002+/-0.002
Treefish	MPA	0.019+/-0.007	0.013+/-0.007	0.007+/-0.004	0.002+/-0.002	0.003+/-0.002	0.009+/-0.004	0+/-0	0.005+/-0.003	0.011+/-0.005	0.004+/-0.003	0.009+/-0.004	0.007+/-0.004	0.013+/-0.005	0.028+/-0.013
	REF	0.013+/-0.006	0.009+/-0.004	0.012+/-0.006	0.012+/-0.005	0.014+/-0.006	0.01+/-0.004	0.003+/-0.002	0.008+/-0.003	0.034+/-0.013	0.011+/-0.005	0.007+/-0.003	0.01+/-0.005	0.017+/-0.005	0.041+/-0.021
Vermilion Rockfish	MPA	0.311+/-0.061	0.31+/-0.063	0.385+/-0.085	0.374+/-0.049	0.434+/-0.056	0.344+/-0.043	0.249+/-0.032	0.421+/-0.054	0.515+/-0.057	0.701+/-0.081	0.771+/-0.094	1.154+/-0.119	1.309+/-0.2	1.681+/-0.203
	REF	0.094+/-0.021	0.115+/-0.032	0.194+/-0.041	0.212+/-0.039	0.145+/-0.036	0.17+/-0.025	0.077+/-0.015	0.151+/-0.024	0.206+/-0.037	0.179+/-0.036	0.147+/-0.028	0.169+/-0.035	0.281+/-0.056	0.267+/-0.062
Yellowtail Rockfish	MPA	0.073+/-0.015	0.035+/-0.007	0.108+/-0.026	0.059+/-0.025	0.078+/-0.021	0.063+/-0.015	0.135+/-0.025	0.151+/-0.033	0.222+/-0.06	0.153+/-0.044	0.062+/-0.014	0.093+/-0.027	0.039+/-0.012	0.137+/-0.047
	REF	0.044+/-0.011	0.019+/-0.004	0.087+/-0.022	0.013+/-0.005	0.039+/-0.014	0.026+/-0.007	0.063+/-0.013	0.131+/-0.029	0.106+/-0.031	0.067+/-0.019	0.041+/-0.012	0.038+/-0.01	0.032+/-0.012	0.043+/-0.012

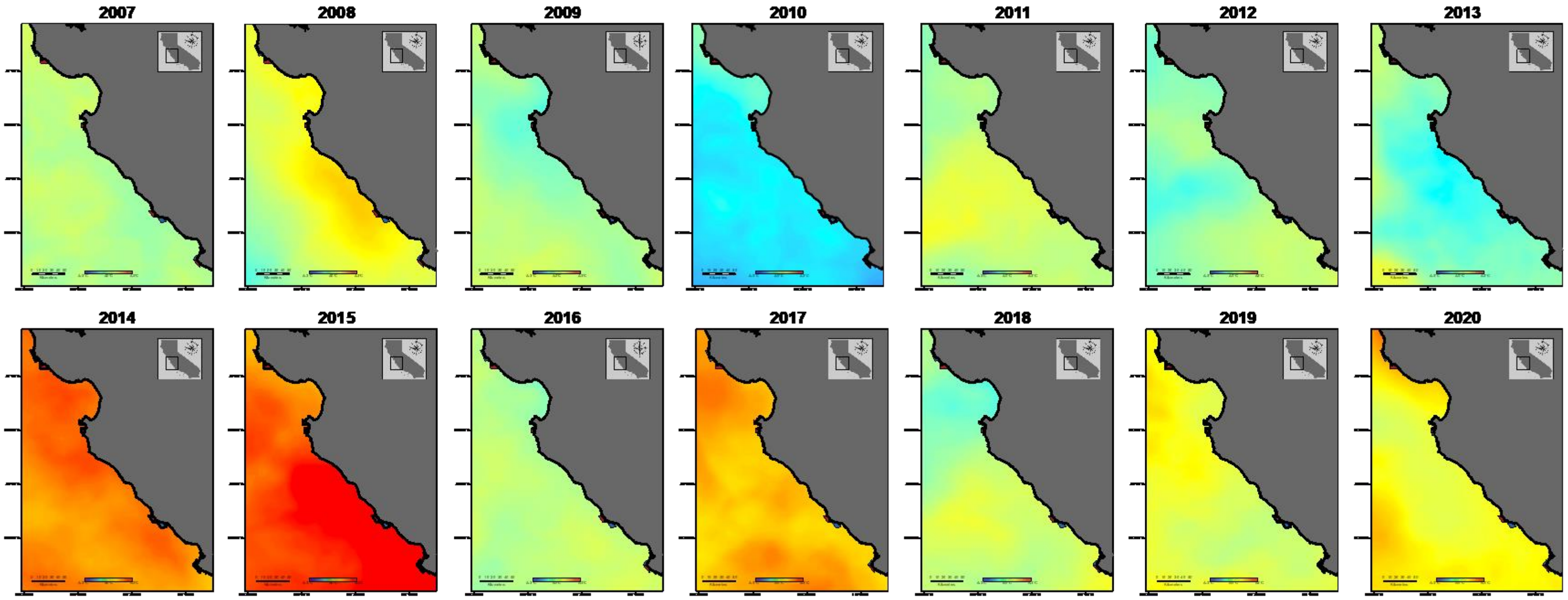

Figure S1: Maps of thermal anomalies in Central California in response to the marine heatwave (MHW). CCFRP MPA and reference sites overlaid with sea surface temperature anomalies for time periods before during and after the 2014-2016 MHW. All temperature anomalies were extracted as monthly means for the month of September for all years of sampling 2007-2020. Data were extracted from the NOAA ERDDAP Multi-scale Ultra-high Resolution (MUR) SST Analysis Anomaly dataset (https://coastwatch.pfeg.noaa.gov/erddap/index.html).

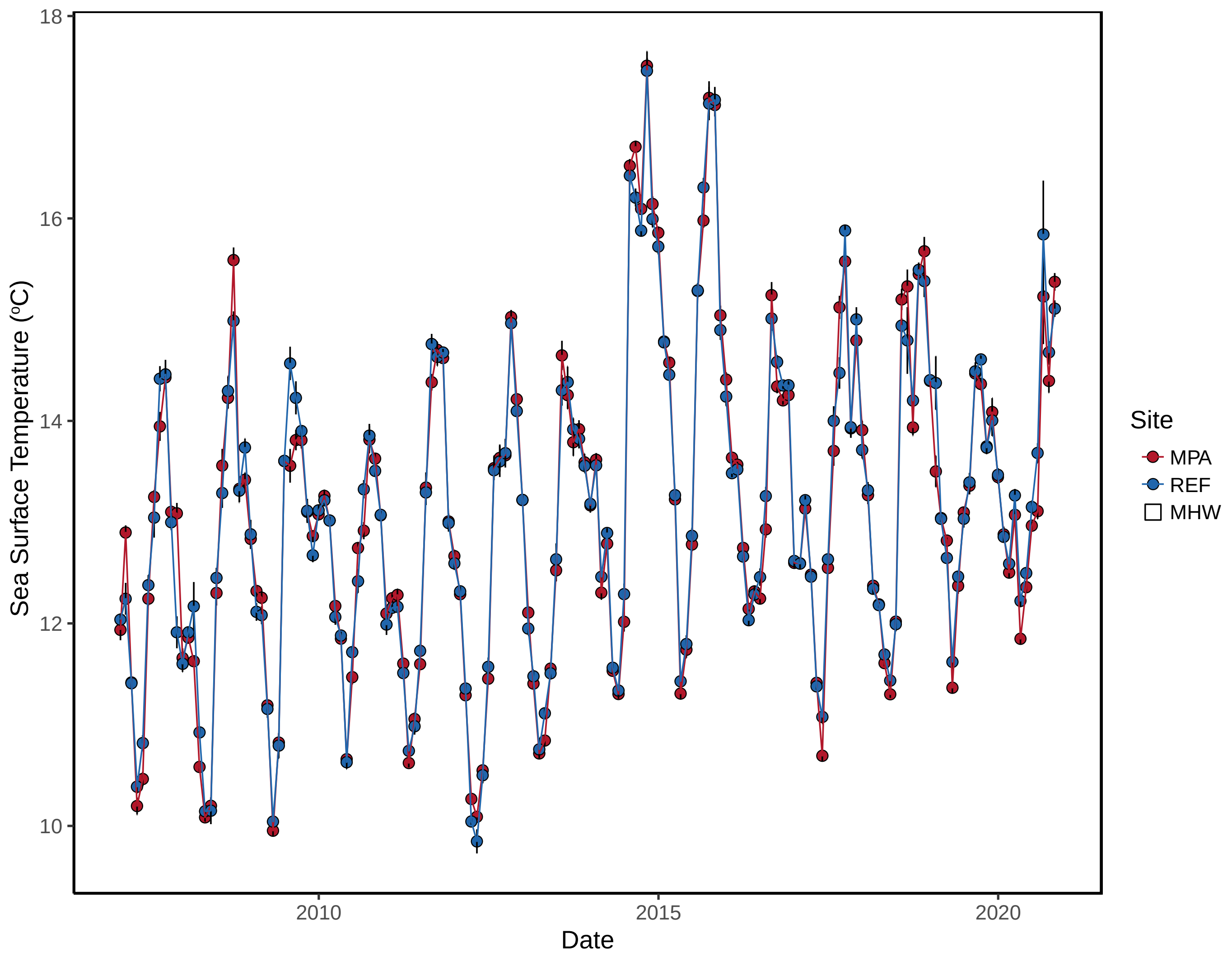

Figure S2. Monthly mean sea surface temperature (SST) over time before (2007-2013), during (2014-2016), and after (2017-2020) the marine heatwave (MHW). Points are monthly means +/- 95% CI

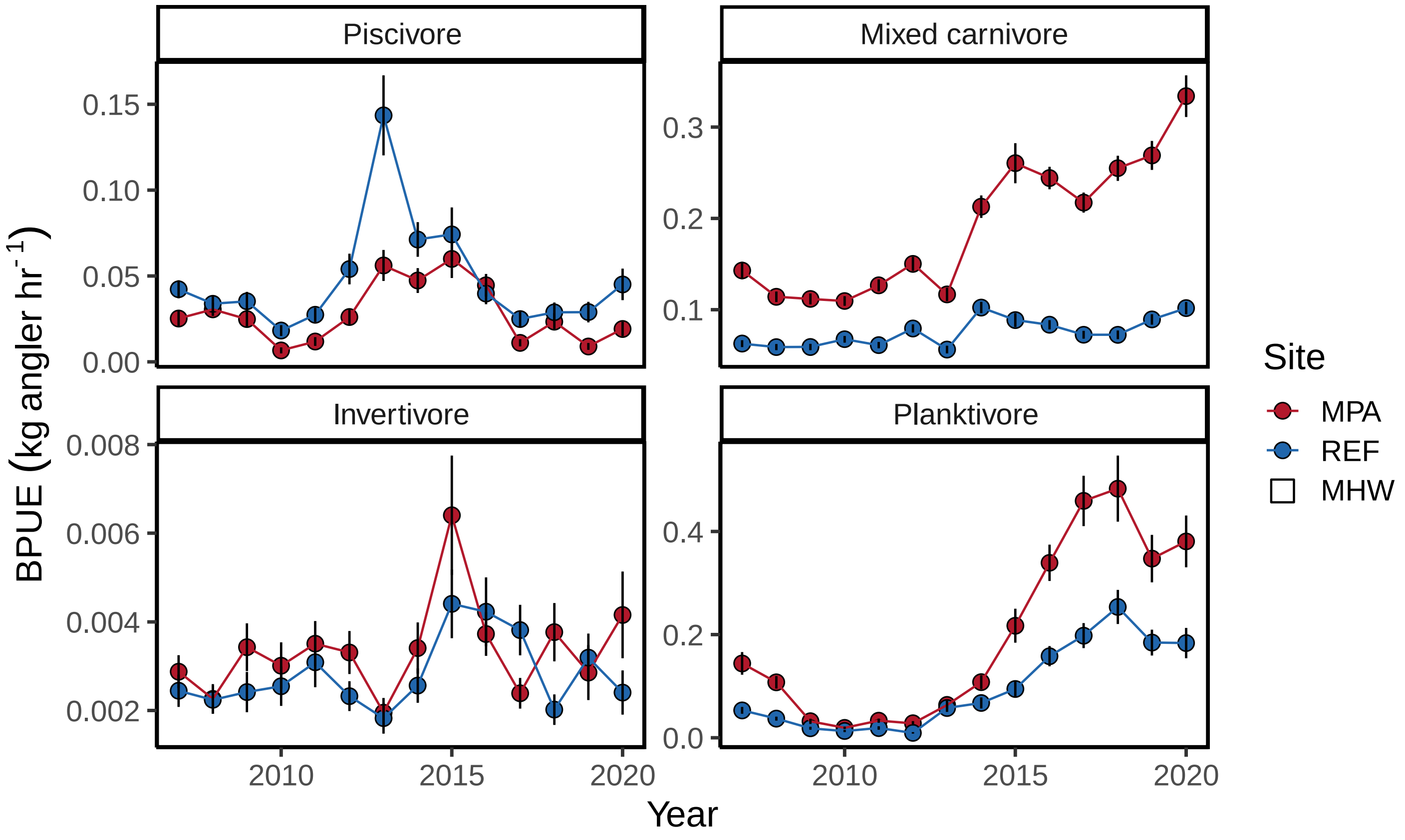

Figure S3. Trends in biomass (BPUE) of trophic groups (piscivores, mixed carnivores, invertivores, and planktivores) at MPA and reference (REF) sites before (2007-2013), during (2014-2016), and after (2017-2020) the marine heatwave. Points are annual means +/- 95% CI

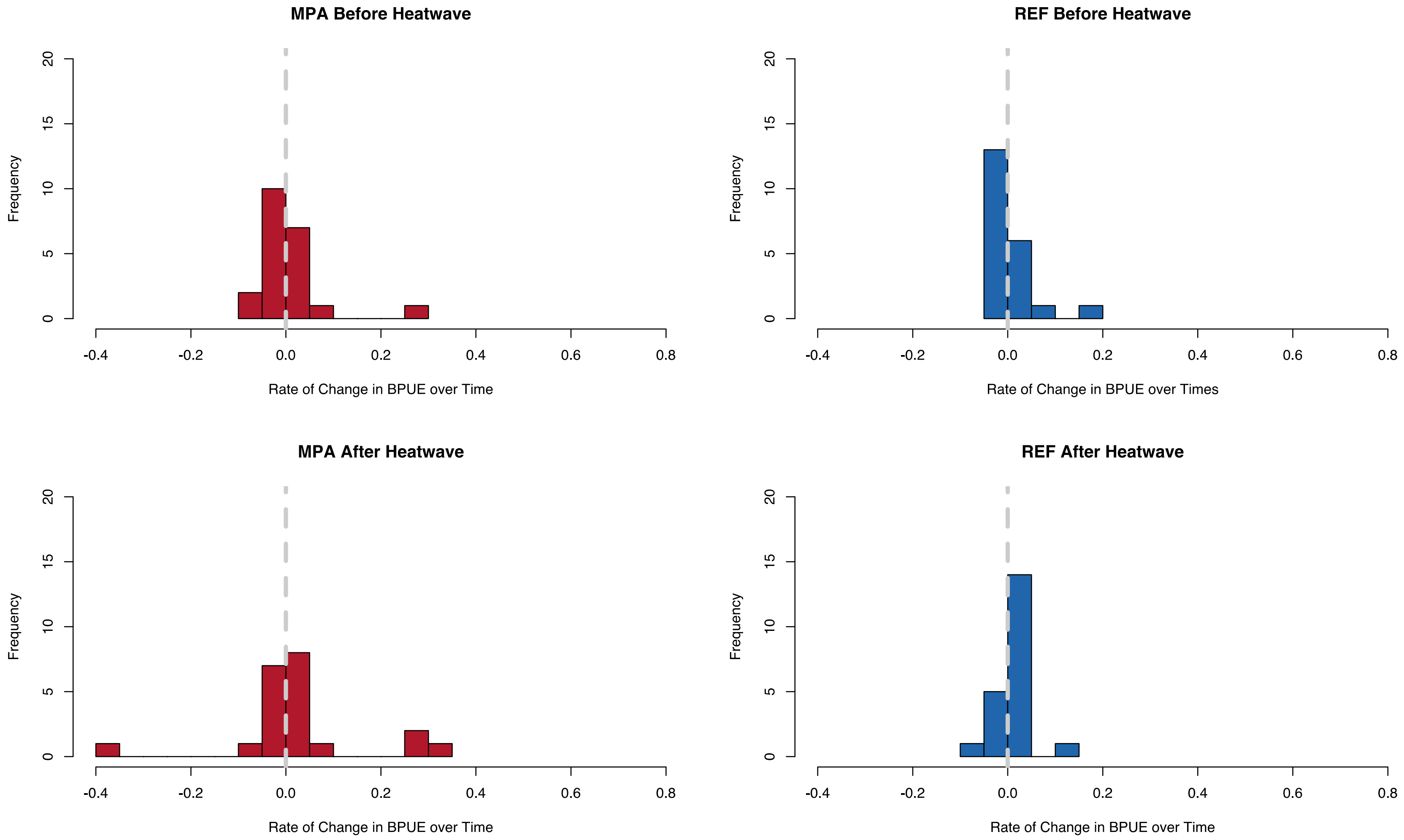

Figure S4. The distribution in the rate of change in biomass (BPUE) of 21 species before (2007-2013) and after (2017-2020) the marine heatwave at MPA and reference (REF) sites.
